# Research trends of acupuncture therapy for painful peripheral nervous system diseases from 2004 to 2023: a bibliometric and meta-analysis

**DOI:** 10.3389/fneur.2025.1510331

**Published:** 2025-03-14

**Authors:** Binke Fan, Yunfan Xia, Yuanyuan Feng, Xilong Yang, Ping Lin, Jianqiao Fang, Zuyong Zhang, Shimin Li

**Affiliations:** ^1^Hangzhou Third Hospital Affiliated to Zhejiang Chinese Medical University, Hangzhou, Zhejiang, China; ^2^The Third Clinical Medical College of Zhejiang Chinese Medical University, Hangzhou, Zhejiang, China

**Keywords:** acupuncture therapy, peripheral nervous system diseases, pain management, peripheral neuropathy, bibliometric analysis, CiteSpace

## Abstract

**Background:**

Peripheral nervous system diseases (PNSD) have represented a major global health burden, leading to significant economic impacts and diminished productivity. This bibliometric analysis was performed to summarize the current research trends and hotspots over the past two decades, aiming to provide a comprehensive perspective for future research.

**Methods:**

All data were sourced from the Web of Science Core Collection (WoSCC) on March 1, 2024, for publications between January 1, 2004, and December 31, 2023. Data visualization and analyses, including descriptive statistics, co-occurrence mapping, and cluster analysis, were performed using CiteSpace (Version 6.1.R6) and Excel 2021.

**Results:**

Our search yielded 678 references, with the annual publication count demonstrating an increasing trend over the past 20 years. The most productive country and institution were China and Kyung Hee University, respectively. Fang Jianqiao, was the most prolific author with the highest publications. Among journals, “*Pain*,” was the most frequently cited, while the top-cited reference was a randomized controlled pilot trial by Lu Weidong in 2020. “Acupuncture” emerged as the keyword with the highest frequency. The meta-analysis indicated that acupuncture was more effective than standard care for pain associated with Diabetic Peripheral Neuropathy [MD = −2.03, 95% CI (−2.86, −1.21), 2 RCTs, 102 participants, *p* < 0.0001].

**Conclusion:**

Our bibliometric review revealed key insights into the focal areas of PNSD research, underscoring the urgent need for continued and targeted high-quality investigations.

## 1 Introduction

PNSD are pathological conditions of the peripheral nerves—comprising nerve roots, ganglia, plexi, and autonomic, sensory, and motor neurons—beyond the brain and spinal cord. These disorders precipitate lower motor neuron dysfunction, which manifests as muscle weakness and paralysis, or impair sensory neurons, causing abnormal sensations or sensory loss. As significant contributors to global morbidity, PNSD include a diverse array of disease types. Notably, peripheral neuropathy manifests a prevalence of 2.4% within the general population, escalating to 8% in individuals aged over 55 (1). The large economic burden of PNSD is pronounced, including direct healthcare costs and indirect costs incurred from productivity loss (2). Despite this, therapeutic strategies are largely palliative rather than curative. Neuropathic pain, a prevalent and debilitating symptom, remains difficult to manage, with pharmacological interventions offering only transient relief and carrying significant long-term risks. Current clinical guidelines thus prioritize non-pharmacological interventions, recognized for their efficacy and potential as alternative treatments (3, 4).

Given these challenges in managing PNSD, this study explored acupuncture, a non-pharmacological approach that has gained international endorsement, as a potential alternative. Acupuncture, a traditional Chinese medical practice, is now widely acknowledged in global healthcare and scientific research communities (5). This modality is effectively employed to manage a variety of conditions including chronic pain, osteoarthritis, seasonal allergic rhinitis, insomnia, and chronic functional constipation, demonstrating substantial therapeutic outcomes (6–10).

Recent years have witnessed a surge in international interest and research into acupuncture, particularly concerning its mechanisms and applications in PNSD. The literature consistently reported on the treatment’s efficacy and safety in managing diverse PNSD conditions such as diabetic peripheral neuropathy, cancer-related symptoms, neuralgia, and herpes zoster (11–14). Despite this, the existing literature remains eclectic and lacks systematic organization. Consequently, our study undertook a bibliometric analysis to map current research trends and identify focal areas within the field.

Bibliometrics, an interdisciplinary field, harnesses quantitative techniques to scrutinize published literature and documents through robust mathematical and statistical methods (15). This methodology merges mathematics, statistics, and philology, underscoring the importance of quantitative analysis (16). Among the plethora of tools available, CiteSpace is extensively employed for bibliometric visualizations, conducting co-citation and co-occurrence analyses.

This study aims to map the current landscape of references in acupuncture therapy for PNSD over the past two decades. It scrutinizes publication years, geographic origins, journals, research domains, contributing authors, prevalent keywords, and employs bibliometric techniques to explore hot topics and emerging trends. The review critically examines the evidence in the published literature regarding the safety and efficacy of acupuncture for PNSD and associated symptoms, comparing it to sham acupuncture or conventional medical therapy. Additionally, the effectiveness of acupuncture across various neuropathic conditions is assessed, where evidence allows.

## 2 Methods

This study adheres to the PRISMA (Preferred Reporting Items for Systematic Reviews and Meta-Analyses) and AMSTAR (A Measurement Tool to Assess Systematic Reviews) guidelines.

### 2.1 Data sources and search strategy

**Figure 1:**
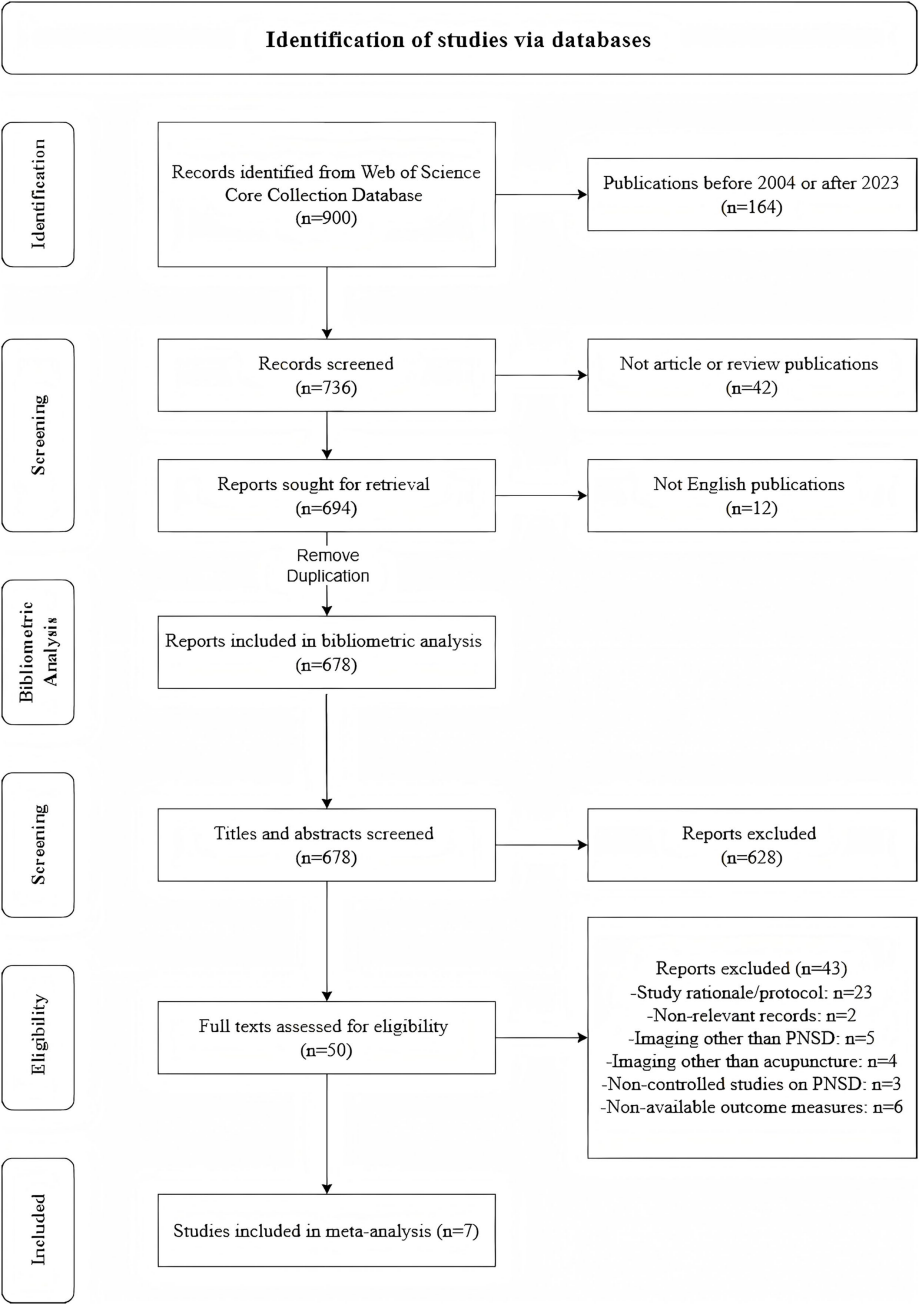
Schematic of data collection and screening.

Data were sourced from the WoSCC on March 1, 2024, encompassing the Science Citation Index Expanded (SCI-EXPANDED), Current Chemical Reactions (CCR-EXPANDED), and Index Chemicus (IC). The investigation utilized search terms “Peripheral Nervous System Diseases” and “Acupuncture Therapy” over the period from January 1, 2004, to December 31, 2023. This search was restricted to English-language articles and reviews. Duplicates were systematically removed. Detailed methodologies, the screening process and the corresponding results are documented in Figure 1 and Supplementary Table S1.

### 2.2 Data extraction and visualization

In this study, we utilized CiteSpace 6.1.R6 to construct a knowledge network map and conducted statistical analysis using Microsoft Excel 2021. Besides, SCImago Graphica (v.1.0.44) was used to analyze countries/regions, institution clustering, and published journals. The CiteSpace configuration was set to analyze data from 2004 to 2023 with a granularity of factor k = 25 and applied Pathfinder pruning to both sliced and merged networks. Nodes in the map represented unique units, with their size indicating occurrence frequency. Nodes exhibiting large dimensions and warm colors highlighted recent frequency surges, whereas those with purple perimeters signaled literature with notable centrality. Line thickness between nodes directly correlated with the level of collaboration or co-occurrence.

### 2.3 Inclusion and exclusion criteria for meta-analysis

#### 2.3.1 Inclusion criteria

1. Studies must be randomized controlled trials (RCTs).
2. Outcomes must include, but are not limited to, measures such as the Symptom Severity Score, Visual Analogue Scale (VAS), Global Symptom Score, or McGill Pain Questionnaire, or employ rigorously defined, study-specific objective criteria to differentiate responders from non-responders.

#### 2.3.2 Exclusion criteria

1. Non-primary research formats, including reviews, conference abstracts, letters, and experimental registrations, are excluded.
2. Non-randomized trials are ineligible.
3. Studies where data are non-extractable or presented in incompatible formats are not considered.

### 2.4 Study quality assessment in meta-analysis

During the meta-analysis of included RCTs, we evaluated the quality of the RCTs using the Cochrane Risk of Bias (ROB) tool, which assesses random sequence generation, allocation concealment, participant and personnel blinding, outcome assessment blinding, incomplete outcome data, selective reporting, and publication bias. Disagreements were resolved through discussion or, if necessary, by consulting a third author.

### 2.5 Statistical analysis

The meta-analysis was performed using Review Manager 5.3. Risk ratios (RR) with 95% confidence intervals (CI) were calculated for dichotomous variables, while mean differences (MD) or standardized mean differences (SMD) were used for continuous variables. Heterogeneity was assessed via the chi-square test and quantified using the *I^2^* statistic. A fixed-effects model was applied when *I^2^* ≤ 50%, whereas a random-effects model was adopted for higher heterogeneity. Sensitivity analyses were conducted to explore potential sources of heterogeneity when substantial variation was detected. Statistical significance was defined as *p* < 0.05.

## 3 Results

### 3.1 Bibliometric analysis

#### 3.1.1 Annual publications

**Figure 2:**
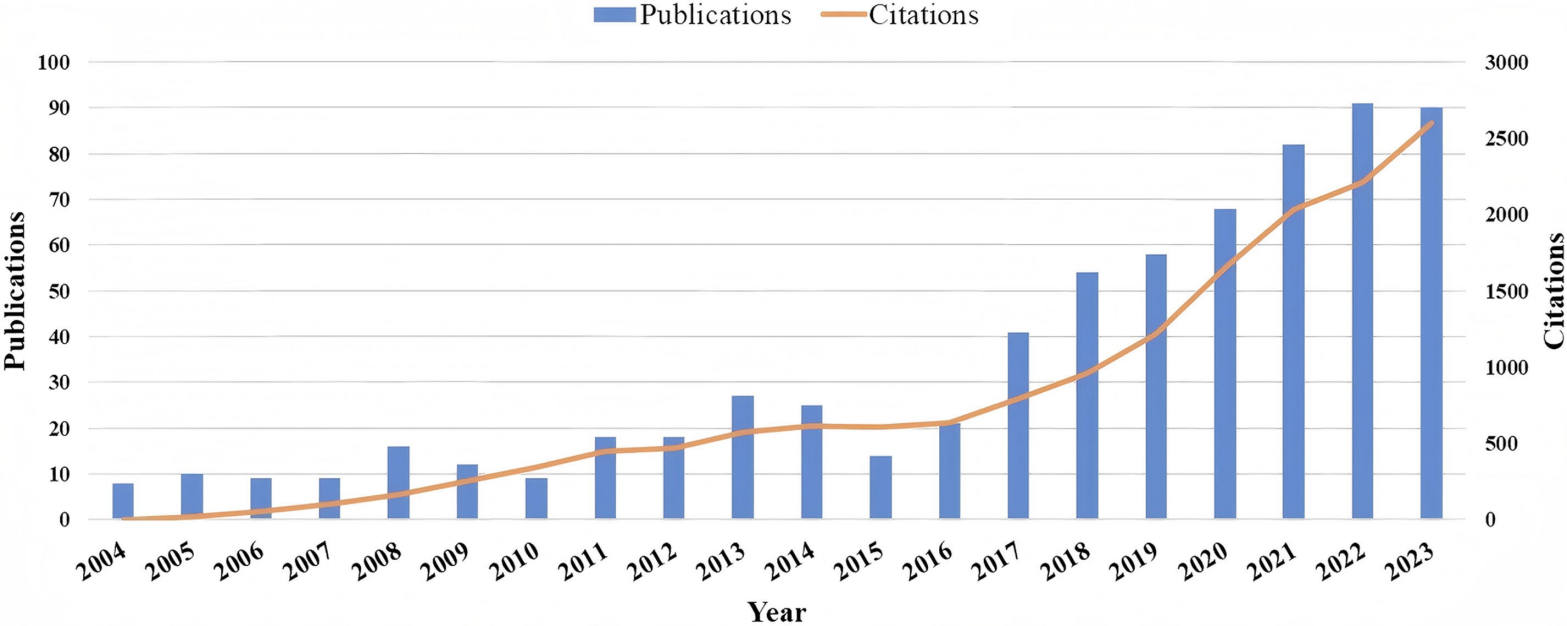
Timeline of publications and citations.

A total of 678 publications were analyzed in this study. Figure 2 depicts a clear upward trajectory in annual publication volume. Initial output between 2004 and 2010 remained at a low level, with annual totals not surpassing 20. A gradual increase was observed from 2011 to 2015, yet the pace of growth was minimal. A marked and continuous escalation in publication numbers commenced in 2016, and culminated in 90 articles, which constituted 13.2% of the total, in 2023. Additionally, we present the Times Cited status for the relevant research. The peak in Times Cited occurred in 2023, with the highest count reaching *N* = 2,603.

#### 3.1.2 Countries

**Figure 3:**
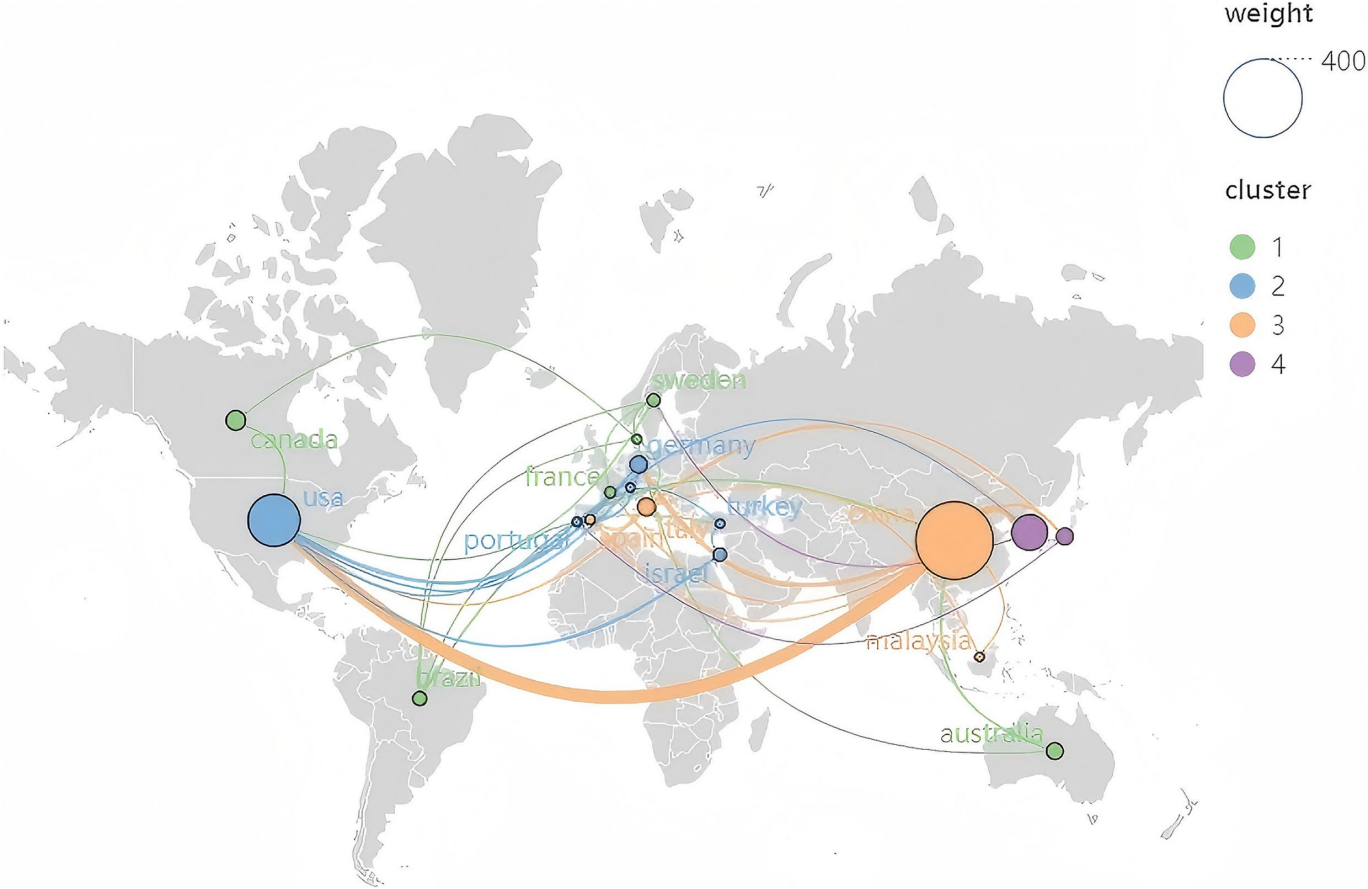
Distribution of countries.

From 2004 through 2023, 41 nations have substantially advanced this area of research, as detailed in Figure 3 and Supplementary Table S2. The countries with the highest publication output included China (355 publications), the United States (160), South Korea (74), England (28), and Canada (22). In terms of centrality, England led with a score of 0.53, followed by Italy (0.38) and Sweden (0.37). Supplementary Figure S1 depicts the cooperative relationships among relevant countries globally, organized into four distinct clusters. Notably, the partnership between China and the USA stands out. The relatively dispersed nature of cooperation among the countries suggests a low level of overall collaborative engagement.

#### 3.1.3 Institutions

**Figure 4:**
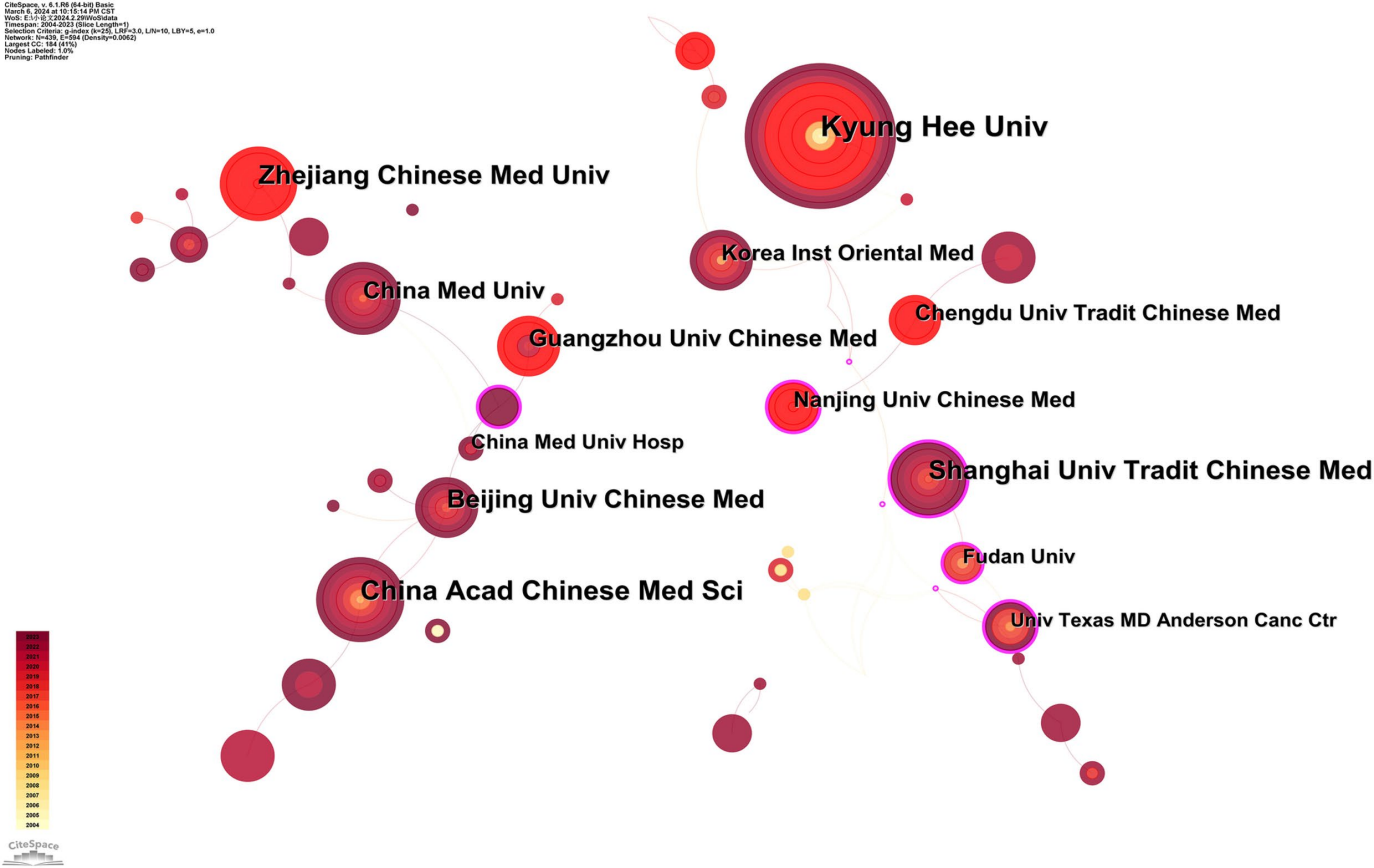
Collaborative map of institutions.

Figure 4 and Supplementary Table S3 show that 439 institutions have actively contributed to this research domain. Leading the publication count were Kyung Hee University, China Academy of Chinese Medical Sciences, and Zhejiang Chinese Medical University, with 40, 30, and 28 publications, respectively. In terms of centrality, the University of Texas MD Anderson Cancer Center, Nanjing University of Chinese Medicine, and Fudan University stood out, each demonstrating significant network centrality values of 0.19, 0.18, and 0.18, respectively. Supplementary Figure S2 presents a network visualization of the cluster analysis for these institutions. The institutions are categorized into 5 cooperative clusters, demonstrating close cooperation among the organizations within each cluster and significant inter-cluster collaboration as well.

#### 3.1.4 Authors

**Figure 5:**
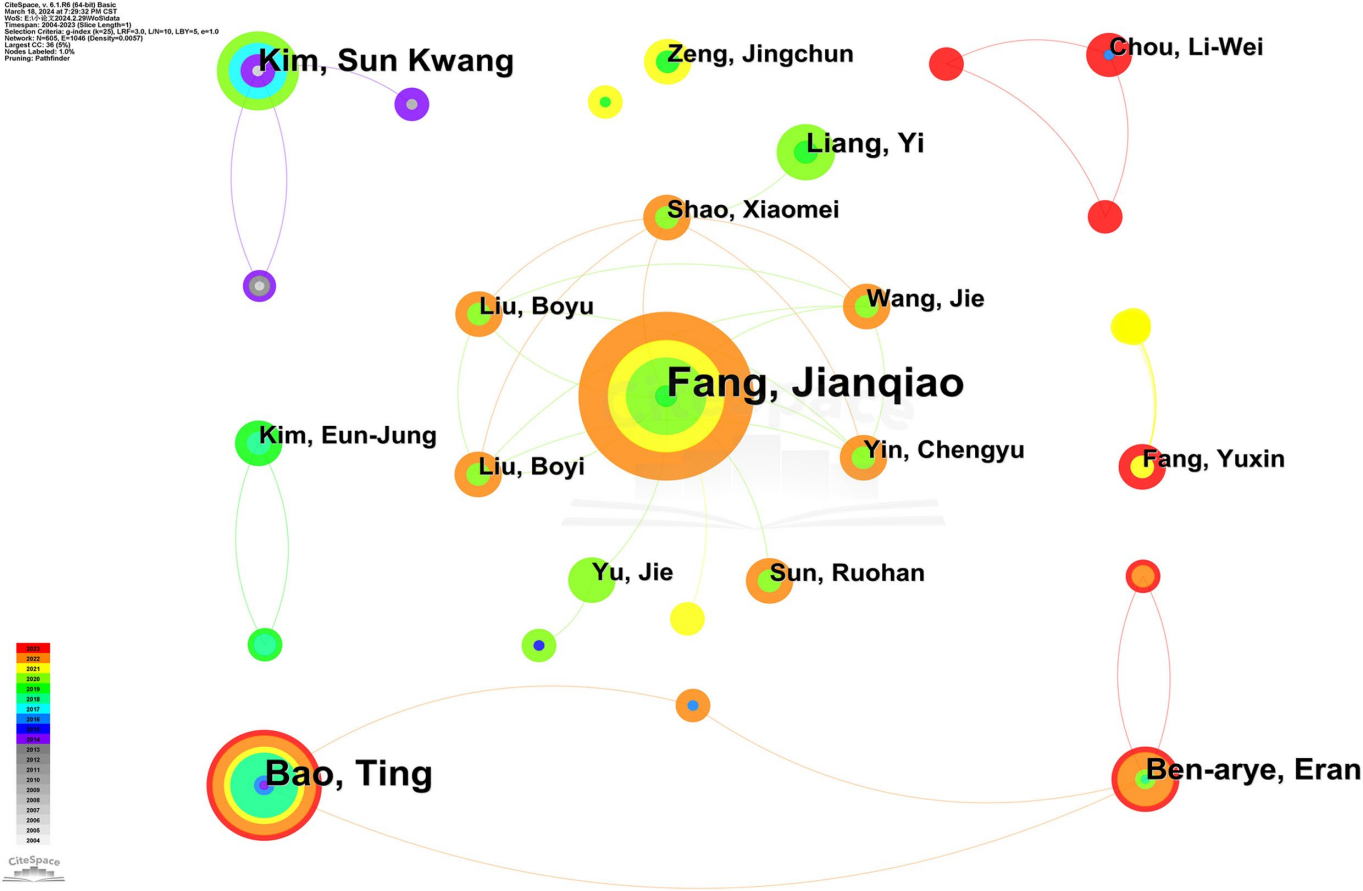
Collaborative map of authors.

In the analysis of acupuncture therapy for PNSD, our study identified 605 researchers, of whom 5 has authored more than five publications, as detailed in Figure 5 and Supplementary Table S4. The size of the nodes in the network analysis corresponds to each author’s publication output. Fang Jianqiao (15) and Bao Ting (10), were identified as the leaders in this field. They were closely followed by Kim Sun Kwang (7), Eran Ben-arye (6), and Liang Yi (5). The data further reveal a distinct lack of collaborative efforts among these key figures. As leading scholars, Fang Jianqiao and Bao Ting have extensively evaluated the analgesic potential of acupuncture through RCTs, to compare its efficacy against placebo interventions like sham acupuncture or conventional therapies, as documented in references. Additionally, both researchers have explored the influence of patient expectations on the therapeutic effectiveness of acupuncture, proposing that psychological factors significantly contribute to treatment outcomes (17, 18). Specifically, Fang Jianqiao has concentrated on neuropathic pain disorders, including complex regional pain syndrome type-I and trigeminal neuralgia (TN) (19, 20). In contrast, Bao Ting has focused on the role of acupuncture in mitigating symptoms of chemotherapy-induced peripheral neuropathy (CIPN) and integrating complementary treatments to enhance the quality of life for cancer patients (21).

#### 3.1.5 Journals

Research on acupuncture therapy for PNSD was broadly disseminated, with 277 journals having published on the topic. Supplementary Table S5 delineates the leading academic journals in this arena. “*Medicine*” emerged as the foremost publisher with 55 articles, followed by “*Evidence-Based Complementary and Alternative Medicine* (26),” “*Acupuncture in Medicine* (22),” “*Journal of Pain Research* (20),” and “*Frontiers in Neurology* (14).”

Supplementary Table S6 lists the top 5 cited journals in the study of acupuncture therapy for PNSD. Notably, “*Pain*” led with 386 citations, while “*Anesthesia and Analgesia*” was distinguished by its centrality, measuring 0.18. These findings underscored the significant influence and exemplarity of the articles published in these journals within the field.

**Figure 6:**
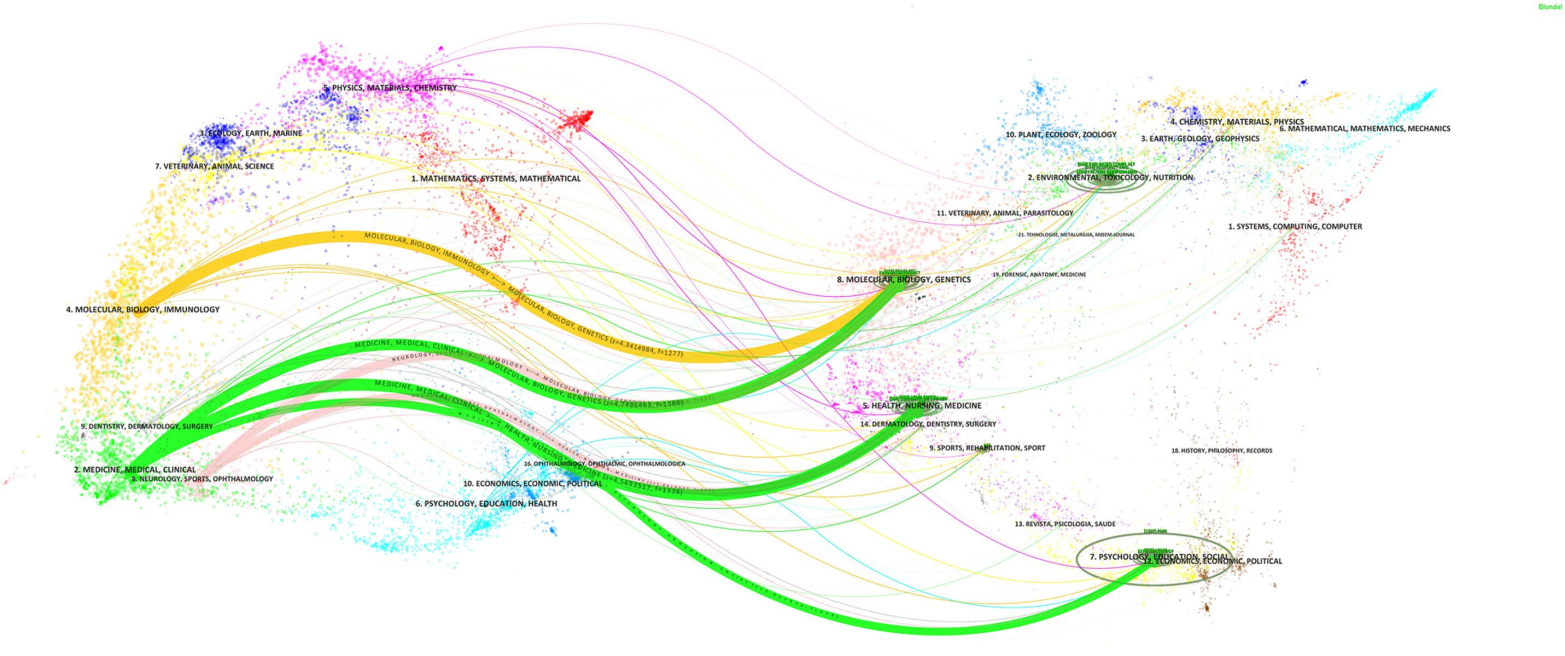
Dual-map overlay of journals.

In this study, CiteSpace was employed to construct a dual-map overlay, elucidating the citation relationships between journals as depicted in Figure 6. The left portion of the overlay maps the citing journals, whereas the right details the cited ones. Notably, the yellow trajectory reveals that articles within “Molecular Biology, Genetics” frequently receive citations from researchers in “Molecular, Biology, Immunology.” Additionally, the green trajectories demonstrate that manuscripts in “Medicine, Medical, Clinical” predominantly reference works across “Molecular, Biology, Genetics,” “Health, Nursing, Medicine,” and “Psychology, Education, Social.” Furthermore, the pink trajectories indicate that outputs in “Molecular, Biology, Genetics” and “Health, Nursing, Medicine” often serve as citation sources for publications in “Neurology, Sports, Ophthalmology.”

#### 3.1.6 Co-cited references

Reference co-citation, the concurrent citation of two or more papers by one or more subsequent publications, serves as a metric to assess the interrelationship among these documents. This measure generally reflects a strong linkage between the cited studies and the corresponding research fields, suggesting that the referenced works are typically of high quality and exert a significant influence within their respective domains. Table 1 list the top five references in terms of co-citation frequency. The study published by Lu Weidong (22) publication had the highest number of co-citations, with Alexandra Dimitrova (23) and Han Xiaoyan (24) works following closely (22).

**Table 1:** The top 5 frequency of citations related to acupuncture therapy for PNSD from 2004 to 2023.

Rank	Reference	Representative author (publication year)	Frequency	Centrality	Journal (IF)
1	Acupuncture for Chemotherapy-Induced Peripheral Neuropathy in Breast Cancer Survivors: A Randomized Controlled Pilot Trial	Lu WD (2020) (22)	34	0.03	Oncologist (5.837)
2	Acupuncture for the Treatment of Peripheral Neuropathy: A Systematic Review and Meta-Analysis	Dimitrova A (2017) (23)	31	0.01	Journal of Alternative and Complementary Medicine (2.381)
3	Acupuncture Combined with Methylcobalamin for the Treatment of Chemotherapy-Induced Peripheral Neuropathy in Patients with Multiple Myeloma	Han XY (2017) (24)	29	0.00	BMC Cancer (4.638)
4	A Randomized Assessor-Blinded Wait-List-Controlled Trial to Assess the Effectiveness of Acupuncture in the Management of Chemotherapy-Induced Peripheral Neuropathy	Molassiotis A (2019) (25)	29	0.00	Integrative Cancer Therapies (3.077)
5	A Phase Iia Trial of Acupuncture to Reduce Chemotherapy-Induced Peripheral Neuropathy Severity During Neoadjuvant or Adjuvant Weekly Paclitaxel Chemotherapy in Breast Cancer Patients	Bao T (2018) (26)	27	0.05	European Journal of Cancer (10.002)

In this trial, Lu Weidong provided robust clinical evidence affirming acupuncture’s effectiveness, safety, and practicability in mitigating symptoms of CIPN. Notably, the study also highlighted acupuncture’s role in not only improving physical neuropathic symptoms but also enhancing survivors’ overall quality of life and functional status.

In a comprehensive meta-analysis conducted by Dimitrova (23), the therapeutic potential of acupuncture for treating peripheral neuropathy across various etiologies was evaluated. This study distinguished between neuropathy types and performing detailed individual subject analyses on conditions such as diabetic neuropathy and Bell’s palsy. The results indicated a significant variability in acupuncture’s effectiveness, contingent upon the neuropathy’s etiological basis.

In a 2017 publication, Han Xiaoyan revealed that acupuncture, used alongside methylcobalamin, could significantly improves outcomes for CIPN in multiple myeloma patients, thereby facilitating the integration of complementary therapies within conventional medical regimes (24). Alexander Molassiotis conducted a RCT in 2019 to underscore acupuncture not merely as a palliative intervention for pain but as a viable treatment strategy for CIPN. His research extended into examining the enduring effects of acupuncture through a methodologically robust, RCT incorporating detailed patient-reported and clinically validated outcome measures (25). A single-arm clinical trial published in 2018 by Bao Ting introduced novel perspectives on the prophylactic potential of acupuncture in managing CIPN, advocating for its use beyond therapeutic applications (26).

**Figure 7:**
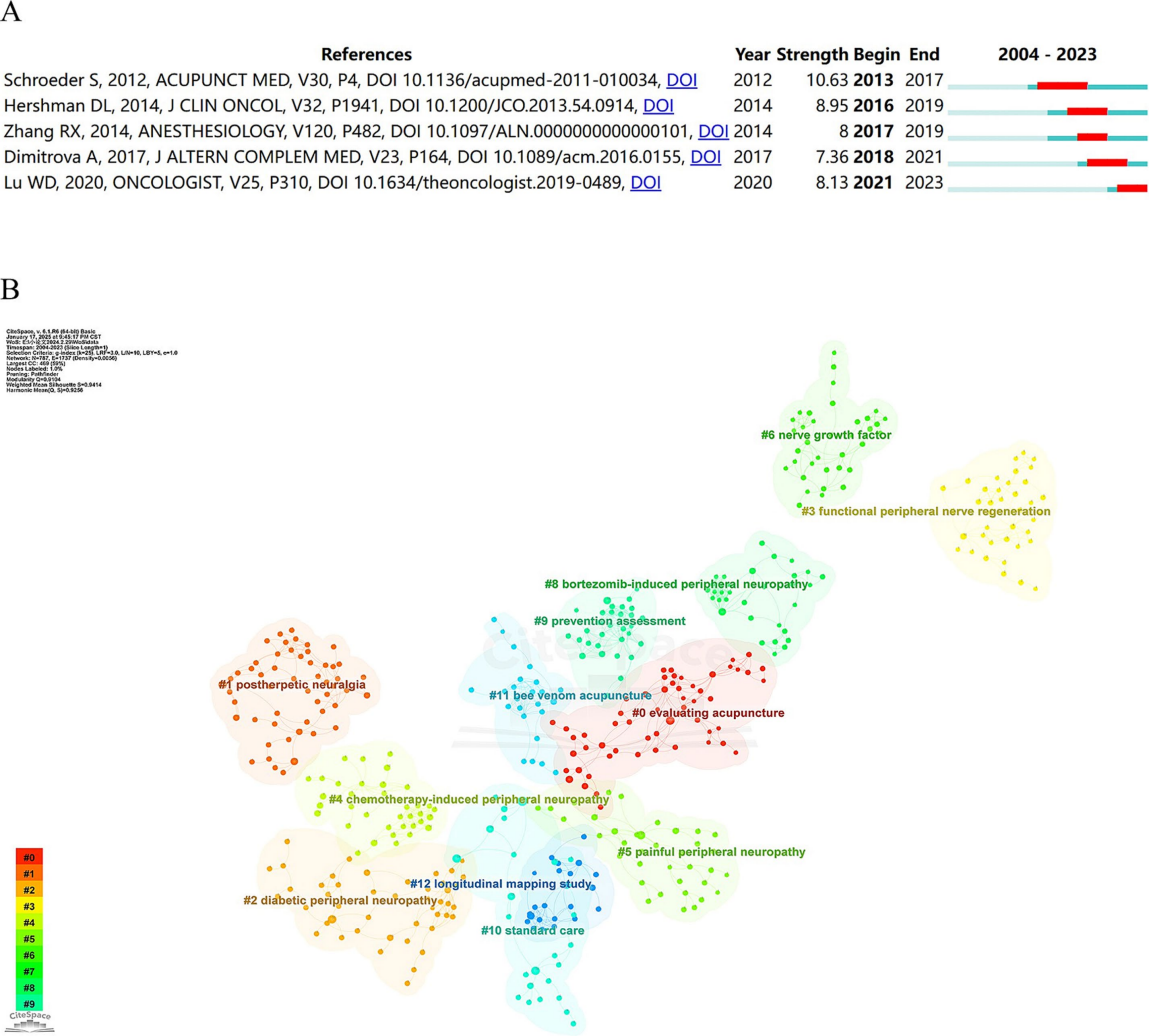
**(A)** Top 5 references with the strongest citation bursts. **(B)** Cluster map of co-cited references.

Citation burst, defined as a substantial increase in citations within a limited timeframe, serves as an indicator of emerging research focal points and potential future directions in a field. Figure 7A displays the five most prominent references exhibiting strong citation bursts, two of which are also among the most frequently co-cited works. Notably, Sven Schroeder et al. (77) had recorded the highest burst strength at 10.63. Moreover, Lu Weidong (22) had attracted considerable attention from the academic community since 2021, a trend that remains ongoing.

To elucidate the structure within the cited reference network, the log-likelihood ratio test (LLR) was utilized to identify clusters based on the keywords from the literature. This approach identified 26 distinct clusters (Figure 7B). Each cluster demonstrated a modularity score of 0.9104 and an average silhouette score of 0.9414, confirming the robustness and high resolution of the cluster graph. Analysis of the timeline view (Supplementary Figure S3) revealed that the primary research areas—namely cluster #1 postherpetic neuralgia (PHN) and cluster #4 CIPN —have emerged as recent research priorities, potentially indicating directions for future investigations.

#### 3.1.7 Keywords

Investigations into prevailing trends and focal points within specific disciplines often rely on the analysis of keywords marked by pronounced centrality and frequency. Supplementary Figure S4 depicts a map of keyword co-occurrence, and Table 2 details the principal keywords according to their frequency and centrality. In the realm of acupuncture therapy for PNSD, the past two decades have seen a consistent prominence of keywords such as “acupuncture,” “pain,” “management,” “neuropathic pain,” and “electroacupuncture (EA).” Keywords with the highest centrality, indicative of their pivotal role in the field, included “acupoint stimulation,” “activation,” “peripheral nerve injury,” “allodynia,” and “electrical nerve stimulation.”

**Table 2:** The top 10 frequency and centrality of keywords related to acupuncture therapy for PNSD from 2004 to 2023.

Rank	Frequency	Keyword	Centrality	Keyword
1	147	Acupuncture	0.19	Acupoint stimulation
2	124	Pain	0.15	Activation
3	99	Management	0.15	Peripheral nerve injury
4	98	Neuropathic pain	0.15	Allodynia
5	81	Electroacupuncture	0.15	Electrical nerve stimulation
6	66	Peripheral neuropathy	0.15	Acupuncture treatment
7	65	Quality of life	0.13	Acupuncture analgesia
8	56	Randomized controlled trial	0.11	Acupuncture
9	48	Systematic review	0.11	Pain
10	46	Mechanism	0.11	Therapy

**Figure 8:**
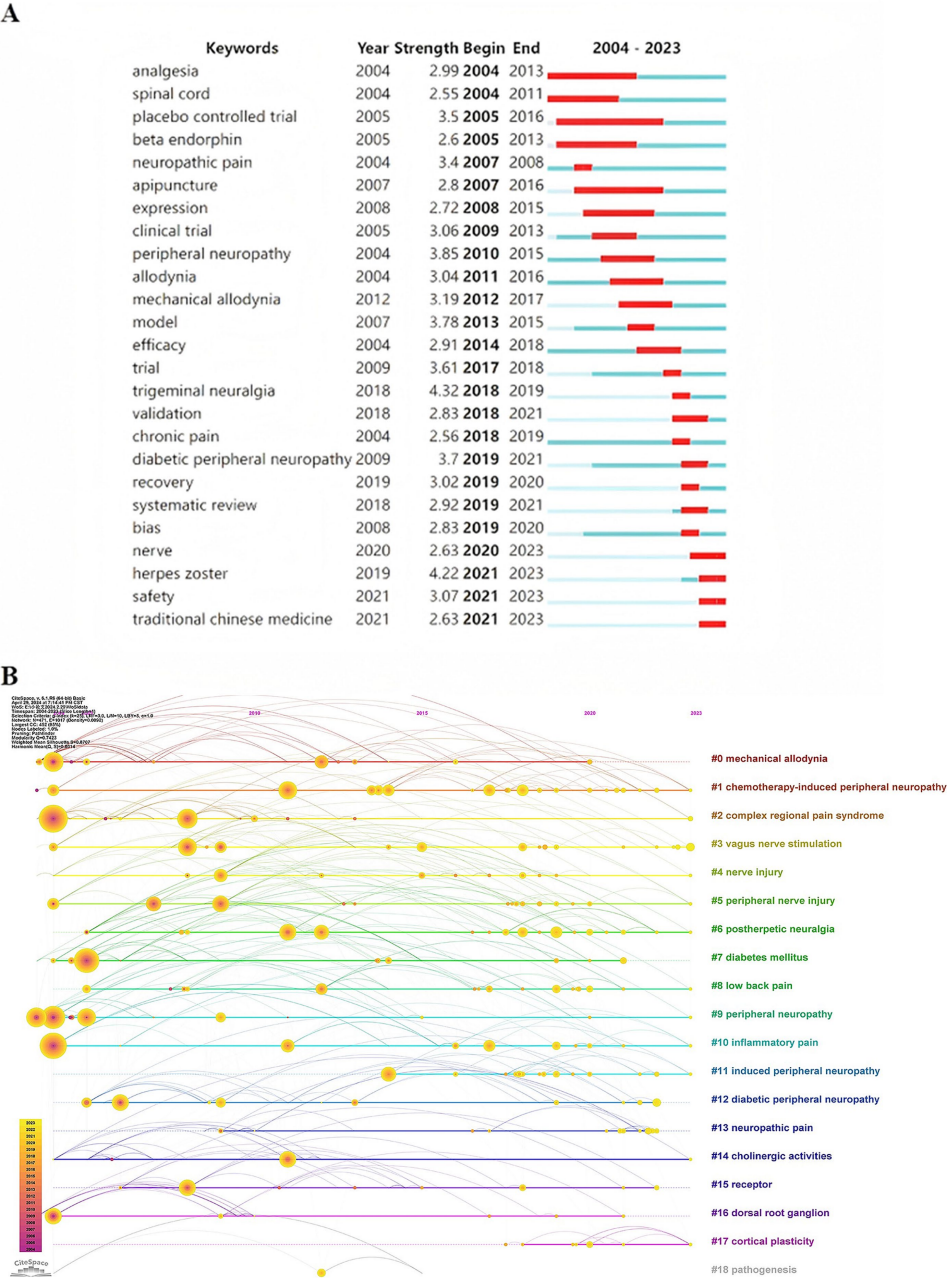
**(A)** Top 25 keywords with the strongest citation bursts. **(B)** Timeline view of keywords co-citation.

In the study, burst words were identified as keywords that were frequently cited within a specific timeframe, underscoring emergent research areas. Figure 8A presents 25 keywords characterized by their initial year of prominence. Among these, “TN” emerged as the most potent, registering a peak strength of 4.32, although its prominence was brief, which lasting only one year. Conversely, “placebo-controlled trials” have demonstrated enduring relevance, originating earlier and sustaining their significance. At present, four keywords— “nerve,” “herpes zoster,” “safety,” and “traditional Chinese medicine”—continue to be identified as areas of ongoing research interest.

On the basis of the co-occurrence of keywords, they were clustered by the algorithm of CiteSpace, and 18 keyword cluster labels were obtained (Figure 8B). In this visualization, nodes represent keywords, arranged chronologically from left to right along the timeline at the top. Multicolored curves illustrate co-citation links between years, with larger nodes or nodes in warm colors signifying high citation frequency, citation bursts, or both. Clusters composed of homogeneous nodes are displayed vertically in descending order of size. Cluster #0, labeled “mechanical allodynia,” is the largest, containing 41 keywords, followed by “CIPN” (34 keywords), “complex regional pain syndrome” (31), “vagus nerve stimulation” (28), and “nerve injury” (28). Although Cluster #0 demonstrates the highest homogeneity, reflected by a silhouette score of 0.918, larger clusters generally exhibit slightly lower homogeneity than smaller clusters. Regarding cluster longevity, Cluster #0 spans 16 years and concludes in 2020, whereas Cluster #1 extends over 20 years and remains active through 2023, the most recent year analyzed.

### 3.2 Meta-analysis

#### 3.2.1 Characteristics of the included RCTs

A total of 7 RCTs related to acupuncture therapy for PNSD were included in this analysis (11, 27–32). All studies were published in English and involved a total of 489 patients, with 249 in the intervention group and 240 in the control group. The sample sizes ranged from a minimum of 32 to a maximum of 121 patients per study. Diagnostic criteria were not reported in one study, Ursini (28). Follow-up times were not reported in three studies: Kong (27), Gao (29), and Stringe (30), while the remaining studies provided this information. The basic characteristics of the included RCTs are summarized in Table 3.

**Table 3:** The characteristics of included RCTs related to acupuncture therapy for PNSD from 2004 to 2023.

Reference	Study period	Region	Type of PNSD	Sample size	Mean age (SD)	Male gender, %	Treatment group interventions	Control group interventions	Course of treatment	Outcome measures
Kong (2020) (27)	2016–2018	USA	Chronic low back pain	T = 59; C = 62	T = 45.76 (11.88); C = 45.58 (12.76)	T = 39.0; C = 46.8	Electroacupuncture	Sham electroacupuncture	6 weeks	Pain Intensity
Ursini (2011) (28)	2006–2008	Italy	Herpes zoster	T = 34; C = 32	T = 67.1 (12.8); C = 65.5 (12.8)	T = 32.3; C = 40.6	Acupuncture	Standard pharmacological treatment	4 weeks	Response rate
Gao (2022) (29)	2019–2021	China	Postherpetic neuralgia	T = 30; C = 30	T = 60.4(9.4); C = 63.0(9.0)	T = 50; C = 53.3	Acupuncture + ultrasound-guided paravertebral nerve block combined	Ultrasound-guided paraspinal nerve block	4 weeks	Response rate
Stringe (2022) (30)	2015–2018	England	Chemotherapy-induced peripheral neuropathy	T = 59; C = 61	T = 61 (8.6); C = 60 (10.9)	/	Acupuncture + standard treatment	Standard treatment	10 weeks	Pain score
Huang (2023) (11)	2016–2019	China	Chemotherapy-induced peripheral neuropathy	T = 16; C = 16	T = 52.0 (13.4); C = 52.0 (13.8)	T = 30.8; C = 46.2	Acupuncture	Sham acupuncture	36 weeks	BPI-SF
Chao (2019) (31)	2015	USA	Diabetic peripheral neuropathy	T = 26; C = 14	T = 61.0(9.8); C = 60.7(11.8)	T = 46.2; C = 57.1	Acupuncture + usual care	Usual care	12 weeks	NRS score
Dietzel (2023) (32)	2019–2021	German	Diabetic peripheral neuropathy	T = 31; C = 31	T = 66.7 (7.6); C = 69.5 (7.2)	T = 80.6; C = 77.4	acupuncture + routine care	Routine care	8 weeks	VAS DPN pain

T, treatment group; C, the control group; BPI-SF, brief pain inventory-short form; NRS, numerical rating scale; VAS, visual analogue scale.

#### 3.2.2 Quality assessment of the included RCTs

**Figure 9:**
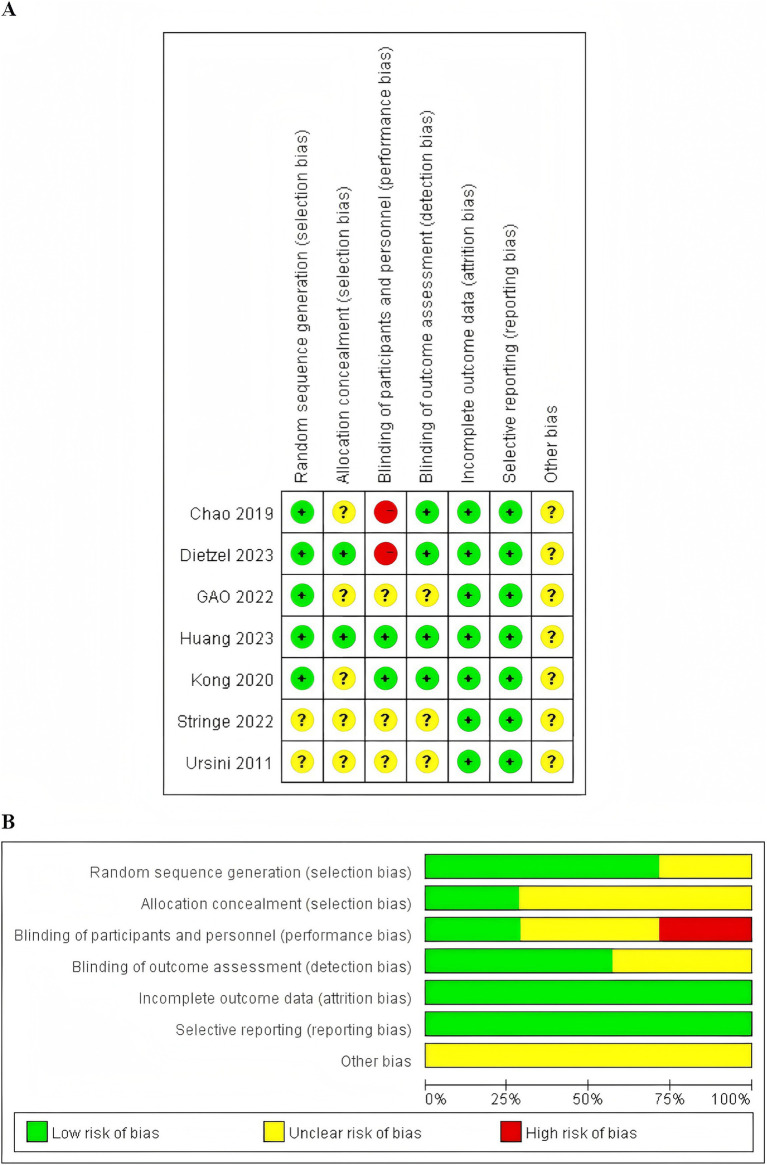
**(A)** Risk of bias of 7 included RCTs. **(B)** Risk of bias summary of 7 included RCTs.

The risk of bias regarding the primary outcome across the 7 RCTs is detailed in Figure 9. Five RCTs utilized the random number table and computer software for sequence generation, indicating a low risk of bias (11, 27, 29, 31, 32). Two RCTs mentioned “random” without specifying details, resulting in an unclear risk of bias (28, 30). One RCT reported using opaque envelopes for sequence concealment (11), and another reported central randomization via telephone control (32), both of which were deemed to have a low risk of bias. Studies not providing this information were considered to have an unclear risk of bias. Two studies reported blinding methods: one applied blinding to patients and primary researchers without compromise (27), and the other employed blinding for participants, evaluators, and statisticians but not for acupuncturists and study coordinators, which was not deemed to introduce bias (11). Thus, the risk of bias for these studies was considered low. Studies not reporting blinding or not blinding both patients and primary researchers were considered to have an unclear or high risk of bias. All studies were assessed as having a low risk of bias regarding incomplete outcome data. Except for one study (27), the remaining RCTs did not report their study protocol. Since all studies had complete registration information and reported all expected outcome measures, including those pre-specified in the published literature, all RCTs were considered to have a low risk of bias. However, due to inadequate reporting on aspects such as random allocation methods, sequence concealment, and blinding implementation, along with a high risk of methodological bias, the overall methodological quality of the included RCTs was not considered high.

#### 3.2.3 Pain intensity of acupuncture for PNSD

**Figure 10:**
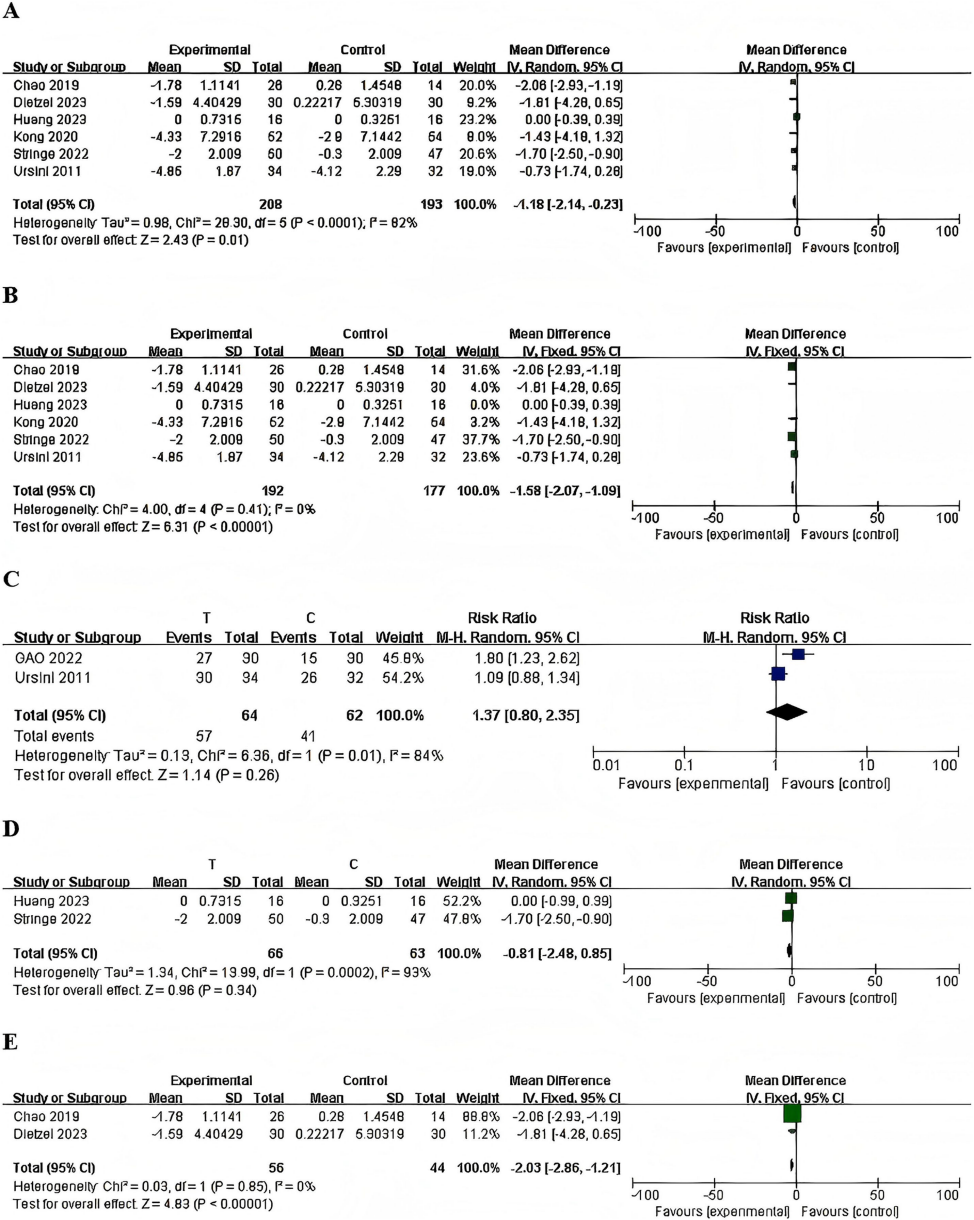
Forest plots of the pain intensity. **(A)** Total, **(B)** sensitivity analysis, **(C)** herpes zoster, **(D)** chemotherapy-induced peripheral neuropathy, **(E)** diabetic peripheral neuropathy.

The meta-analysis offers a comprehensive synthesis of the data, highlighting key trends and patterns observed across the included studies. The pooled effect sizes demonstrated that acupuncture significantly alleviated pain intensity associated with PNSD, with a mean difference (MD) of −1.18 (95% CI: −2.14, −0.23; *p* = 0.01) (Figure 10A). Heterogeneity was evaluated using the *I^2^* statistic, revealing substantial variability. Subgroup analyses further examined potential moderators, providing insights into the heterogeneity of study characteristics, as outlined below.

##### 3.2.3.1 Sensitivity analysis

The observed heterogeneity in the analyses can likely be attributed to variations in study designs, populations, and intervention protocols. To address this, we employed a Leave-One-Out Analysis, systematically excluding each study from the meta-analysis to evaluate its effect on the overall outcome. The sensitivity analysis identified a single study (11) as having a substantial impact on heterogeneity. Exclusion of this study led to a significant reduction in heterogeneity (*I^2^*), decreasing from 82 to 0%, indicating its central role in driving the observed variability. The pooled effect size remained robust (from *p* = 0.01 to *p* < 0.0001, with a new MD = −1.58, 95% CI [−2.07, −1.09]), reinforcing the stability of the analysis’ main conclusions, which are not contingent on the inclusion of this study (Figure 10B). These results prompted the exclusion of the study from the final analysis to enhance the reliability and interpretability of the findings.

##### 3.2.3.2 Subgroup analysis

###### 3.2.3.2.1 Chronic low back pain

One RCT reported that electroacupuncture, compared with sham electroacupuncture, showed no significant difference in pain score changes (27). [MD = −1.43, 95% CI (−4.18, 1.32), one RCT, 106 participants, *p* = 0.31].

###### 3.2.3.2.2 Herpes zoster

The random-effects model meta-analysis revealed no significant differences in herpetic zoster treatment outcomes between the acupuncture therapy group and the control group (28, 29). [RR =1.37, 95% CI (0.80, 2.35), 2 RCTs, 126 participants, *p* = 0.26] (Figure 10C).

###### 3.2.3.2.3 Chemotherapy-induced peripheral neuropathy

The random-effects model meta-analysis indicated no significant differences in treatment outcomes between the acupuncture therapy group and the control group for CIPN (11, 30). [MD = −0.81, 95% CI (−2.48, 0.85), 2 RCTs, 152 participants, *p* = 0.34] (Figure 10D).

###### 3.2.3.2.4 Diabetic peripheral neuropathy

In the comparison between acupuncture combined with usual care and usual care alone, the results indicated that acupuncture made significant difference in pain associated with Diabetic Peripheral Neuropathy (31, 32) [MD = −2.03, 95% CI (−2.86, −1.21), 2 RCTs, 102 participants, *p* < 0.0001] (Figure 10E).

#### 3.2.4 Acupuncture safety, adverse events

The included studies exhibited considerable variation in the definition and monitoring of adverse events. Overall, acupuncture treatment was deemed safe, with no serious adverse events reported. One study documented 16 minor adverse events in 11 participants, including tingling, ache/pain, bruising, and minor bleeding, none of which required intervention or withdrawal from the trial (30). Another study reported a total of 43 adverse events associated with acupuncture out of 660 treatments administered; these included 18 small hematomas at single needling sites and seven instances of transient paraesthesia, with one case leading to a patient’s withdrawal from the intervention group (32).

#### 3.2.5 Publication bias

**Figure 11:**
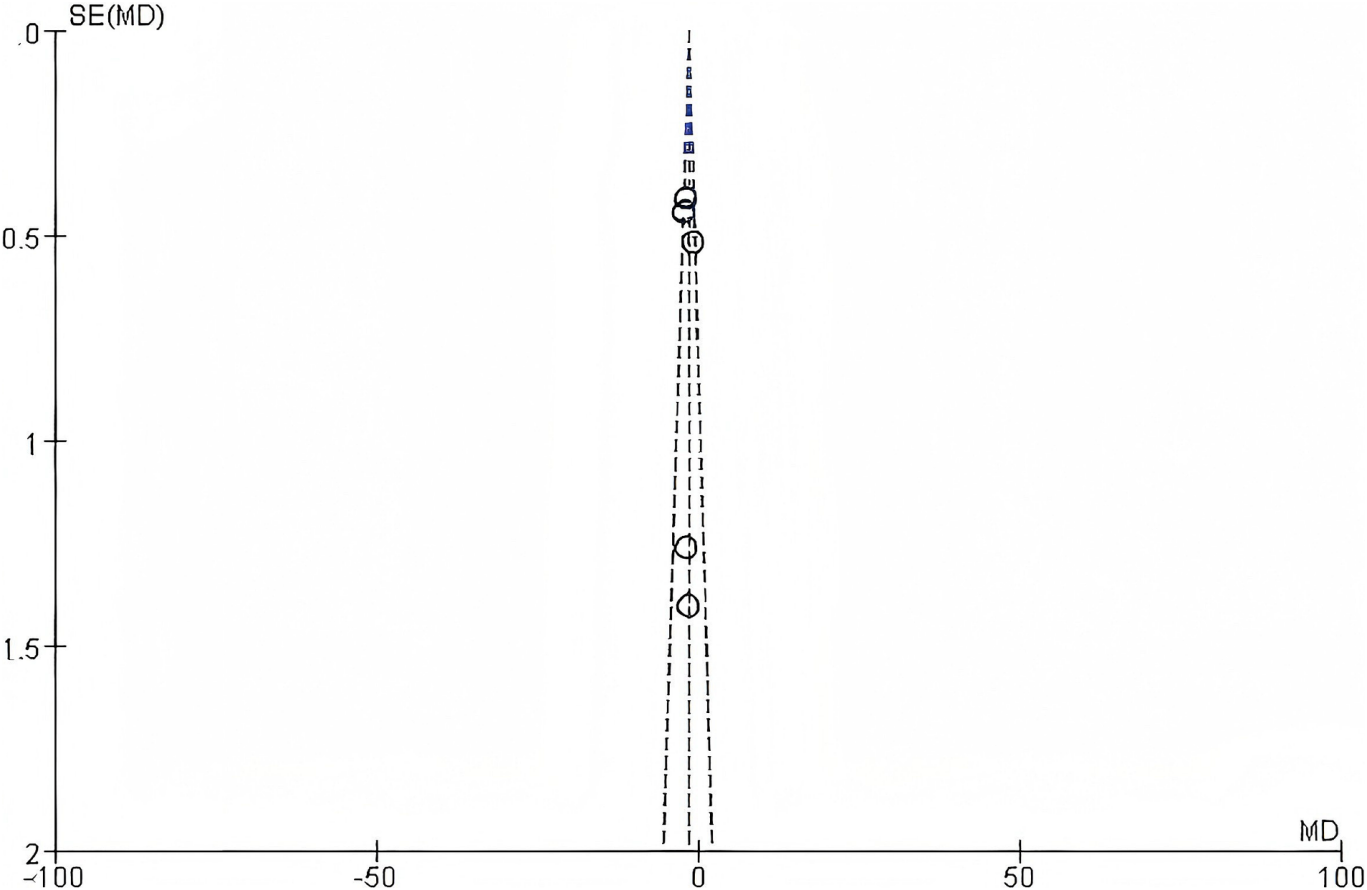
Funnel plot.

Funnel plots were generated to assess the potential for publication bias among the seven RCTs. The distribution of data points in the funnel plots appeared largely symmetrical (Figure 11).

## 4 Discussion

### 4.1 General information

This comprehensive bibliometric review illustrated a consistent uptick in the volume of publications on acupuncture therapy for PNSD spanning two decades. A marked increase in research output is evident starting around 2016, highlighting a growing interest in the therapeutic applications of acupuncture for PNSD in recent years. For the rising domestic incidence of PNSD (33), China, the United States, and South Korea emerged as principal contributors with notable publication outputs, contrasted by England, Italy, and Sweden, which demonstrated pivotal roles through their central positions in the literature network. Kyung Hee University, the China Academy of Chinese Medical Sciences, and Zhejiang Chinese Medical University were the leading institutions in terms of publication volume. Notably, Kyung Hee University was a pioneer in this field, initiating research as early as 2004. Although Zhejiang Chinese Medical University began its research efforts later, it has recently experienced a significant surge in literature output. Among individual researchers, Fang Jianqiao and Bao Ting were distinguished by their prolific contributions, yet the absence of centrality among authors suggested the lack of a stable collaborative network. A total of 71 research areas were identified among the publications included in this study. The top five most represented research areas are Integrative Complementary Medicine, Neurosciences, Medicine General Internal, Clinical Neurology, and Oncology. In terms of journal influence, “*Pain*” and “*Anesthesia and Analgesia*” stood out for their citation frequency and centrality, respectively, underscoring their influence in shaping the discourse within this field.

In current researches, meta-analyses and RCTs serve as the primary methodologies, focusing on the mechanisms and clinical implications of acupuncture. Over time, there has been a trend toward higher methodological rigor, including greater use of randomization, blinding, and standardized outcome measures. Nevertheless, the overall quality of extant research remains inadequate, with the evidence supporting the efficacy and safety of these interventions being limited. Significant challenges in this domain encompass small sample sizes, diverse study designs, and an absence of standardized protocols for acupuncture. Rigorous, large-scale RCTs are critical to delineate the specific benefits of acupuncture in both prophylaxis and treatment of PNSD.

### 4.2 Research hotspots

#### 4.2.1 Acupuncture therapy in pain management

Acupuncture—a treatment with minimal side effects—has increasingly been adopted in global pain clinics for managing both acute and chronic pain scenarios. Extensive research corroborates its efficacy and safety in addressing diverse pain conditions (34–36). PNSD typically manifests with painful paresthesias, posing significant therapeutic challenges. Recent studies have rigorously assessed the therapeutic efficacy and safety of acupuncture in alleviating pain related to PNSD (37–40). Empirical data from multiple studies endorse acupuncture’s effectiveness in diminishing pain severity and minimizing functional disruptions, with minimal adverse events reported. Notably, clinical findings demonstrated that acupuncture not only diminishes pain across various time frames but also offers sustained pain management benefits (41, 42). Furthermore, emerging research underscored the potential of early acupuncture interventions to curb symptom progression and its synergistic benefits when combined with methylcobalamin in combating CIPN (24, 26).

Meanwhile, recent studies have provided mixed results regarding the efficacy of acupuncture for pain management. For instance, one investigation found no significant differences in pain relief between true acupuncture and sham groups at the 12-week mark (43). Similarly, another study is unable to confirm the benefits of acupuncture in reducing neuropathic pain or enhancing quality of life (44). The objective evaluation of acupuncture is inherently challenging due to the variability in practice techniques and the practitioner-dependent nature of treatment modalities. Furthermore, the effectiveness of acupuncture varies widely among different etiologies of PNSD (45, 46). The lack of a standardized placebo control also complicates the differentiation between placebo effects and treatment efficacy (47).

#### 4.2.2 Acupuncture therapy for specific diseases

##### 4.2.2.1 Chemotherapy-induced peripheral neuropathy

Research consistently highlights acupuncture’s efficacy in mitigating symptoms associated with CIPN, including significant reductions in neuropathic pain and paresthesia (24, 26, 48). Notably, RCTs have demonstrated marked improvements in sensory deficits, such as sensation loss and numbness (25). Observational studies further link these symptomatic improvements to enhanced nerve conduction, implying that acupuncture may promote nerve regeneration (49, 50). Importantly, significant enhancements in patient quality of life and reductions in neuropathy severity have been documented (51–53). Nevertheless, the efficacy of acupuncture varies due to differing methodologies, such as the choice between manual acupuncture (MA) and EA (54). Moreover, transcutaneous electrical nerve stimulation (TENS) has achieved notable pain relief in early treatment stages in a limited patient group, yet the intrinsic partially self-healing nature of CIPN necessitates further validation through rigorous RCTs (55).

##### 4.2.2.2 Postherpetic neuralgia

PHN emerges as a chronic pain syndrome following herpes zoster infection, influenced by several risk factors including advanced age, immunosuppression, and autoimmune disorders such as systemic lupus erythematosus and diabetes mellitus, alongside recent physical trauma (56–58). Comprehensive research has established the efficacy of acupuncture in mitigating various forms of neuropathic pain, such as persistent spontaneous, paroxysmal, and mechanically evoked pain, the latter reflecting pathological amplifications of responses to both benign and noxious stimuli (59, 60). Beyond conventional acupuncture, alternative modalities exhibit distinct therapeutic indications and mechanisms, proving beneficial for PHN management. Notably, the use of filiform fire needle acupuncture coupled with mild moxibustion outperforms gabapentin in providing rapid analgesia, reducing pain swiftly with fewer side effects and at a lower cost. RCTs have demonstrated that combining EA at Jia Ji acupoints with moxibustion and intermediate frequency therapy effectively alleviates pain and anxiety in PHN sufferers. Additionally, evidence supports the effectiveness of collateral-pricking and bloodletting cupping combined with EA in enhancing pain relief, sleep quality, and overall therapeutic outcomes in PHN patients (61).

##### 4.2.2.3 Trigeminal neuralgia

Acupuncture is increasingly recognized as a therapeutic intervention for TN. Empirical evidence underscores its role in mitigating the distinctive, severe pain often described as intermittent, electric shock-like sensations affecting the trigeminal nerve divisions (62, 63). A longitudinal analysis supports acupuncture’s therapeutic potential, particularly its analgesic benefits for TN and associated myofascial pain (64). Moreover, clinical trials have documented enhancements in cognitive function and overall quality of life for patients undergoing acupuncture treatment for this neuralgia (65). Additionally, meta-analytical findings suggest that acupuncture provides beneficial effects in managing persistent TN with a favorable safety profile, although the evidence is limited by the methodological shortcomings of the included RCTs (42).

### 4.3 Research frontiers

#### 4.3.1 Focus on patient-reported outcomes

Patient-reported outcomes, such as reductions in pain and improvements in quality of life, were consistently documented across numerous studies, emphasizing the therapeutic potential of acupuncture. Future research should prioritize the development of personalized approaches by aligning treatment protocols with individual symptomatology, pain severity, and patient preferences. The integration of pharmacological and non-pharmacological strategies—including acupuncture, moxibustion, and electroacupuncture—offers a promising avenue to optimize clinical outcomes and advance holistic care paradigms (59, 60).

Besides, there is an urgent need for studies that elucidate the precise mechanisms by which acupuncture modulates PNSD. Determining whether the observed benefits are attributable to the acupuncture itself, placebo effects, or a combination thereof is crucial. Despite the ongoing debate surrounding its placebo effects, current evidence does not justify excluding acupuncture as a treatment option under suitable conditions, particularly considering accessibility and cost factors (66).

#### 4.3.2 Cost-effectiveness analysis

Economic evaluation, particularly cost-effectiveness analysis (CEA), is an essential yet often underappreciated aspect of assessing the feasibility of integrating acupuncture interventions into clinical practice. Such evaluations involve analyzing therapeutic benefits and patient outcomes in relation to the associated costs. Incorporating CEA into acupuncture research provides a more comprehensive understanding of the practicality and economic viability of acupuncture across diverse healthcare settings. For example, CEA can help determine whether acupuncture is a cost-effective alternative to conventional treatments in primary care or if it offers value for money in specialized pain management clinics (67, 68). These insights are crucial for informing policy development, clinical practice guidelines, and for guiding both patients and healthcare providers toward the most appropriate and cost-effective treatment options. The feasibility of conducting CEA in acupuncture research is well-established. Several studies have successfully applied economic evaluation methods to acupuncture interventions (69, 70). While this study does not include a cost-effectiveness analysis, we recognize its importance and recommend that future research incorporate economic evaluations to assess the feasibility of acupuncture in various healthcare environments.

Additionally, research funding is a key determinant in guiding academic inquiry and driving innovation. Our analysis observed a predominance of government-sponsored projects, with limited insight into the role of private organizations due to data constraints. This gap underscores the importance of developing more robust datasets that encompass the range of funding sources. Subsequent studies should prioritize the collection of such data to elucidate how funding patterns influence the therapeutic strategies.

#### 4.3.3 Implications for future research

Acupuncture is a globally practiced therapy, and the increased funding for clinical acupuncture studies has facilitated the generation of high-quality primary clinical evidence (71). This study employs CiteSpace visualization software to objectively examine key trends in acupuncture therapy for PNSD from 2004 to 2023. By integrating recent advancements in the field, we aim to delineate the underlying factors that shape research trajectories.

To ensure data consistency and quality, only English-language publications were included in this analysis. However, this approach may introduce language bias by excluding potentially relevant studies published in other languages. Future research should incorporate studies from diverse linguistic sources to mitigate this bias. Moreover, the small sample sizes and significant variations in research designs across current studies highlight the urgent need for large-scale, high-quality RCTs and systematic evaluations. Future studies should seek to enhance the number of RCTs by refining inclusion criteria and conducting additional rigorous trials. Furthermore, a notable limitation of this review lies in the methodological heterogeneity of acupuncture interventions across included studies. Critical parameters such as acupoint selection rationale, needling techniques, and treatment duration were inconsistently reported, preventing subgroup analyses to disentangle their effects. This reflects a broader challenge in acupuncture research, where personalized protocols often prioritize clinical flexibility over standardization. Future research endeavors should concentrate on ameliorating methodological limitations by implementing stringent blinding of subjects and investigators, standardizing acupuncture techniques, and adopting validated, objective evaluation metrics (72). Evaluating the long-term efficacy of acupuncture relative to established pharmacological therapies (73), and investigating the optimal frequencies and combinations of acupuncture interventions are paramount (74–76).

Additionally, with an enhanced understanding of the various types and specific symptoms of PNSD, future research could increasingly focus on the efficacy of acupuncture in addressing sensory impairments (e.g., numbness, paresthesia) and improving quality of life outcomes. Subsequently, further explorations into the specific mechanisms and therapeutic effects of MA, EA, moxibustion, and other alternative therapies, are warranted. Lastly, with the advancement of precision medicine and personalized treatment approaches, future research should focus on customizing treatment plans to the specific conditions of patients.

## 5 Strengths and limitations

In this study, we combined bibliometric analysis and meta-analysis to explore both the evolution of research trends and the statistical synthesis of outcomes in the field of acupuncture therapy for PNSD, providing a comprehensive visualization of key reference data and a critical assessment of the prevailing research paradigms and emerging frontiers. Moreover, the findings of this study may have significant implications for the development and update of clinical practice guidelines in acupuncture. Specifically, our analysis indicates that acupuncture significantly reduces pain intensity associated with PNSD, particularly in Diabetic Peripheral Neuropathy. However, the treatment effects were not identical, which may inform recommendations for acupuncture therapy selection in different types of PNSD. As acupuncture continues to gain recognition in clinical settings, these findings should be considered to enhance evidence-based practices and guide practitioners in making informed treatment decisions.

This work sought to facilitate a multifaceted understanding of the current developments and to pinpoint areas ripe for future research. Nonetheless, several limitations warranted attention. Firstly, this analysis was confined to English-language literature published between 2004 and 2023, sourced exclusively from the Web of Science Core Collection (WoSCC). While this database is widely recognized for bibliometric studies due to its citation indexing and journal coverage, the exclusion of other databases (e.g., PubMed, EMBASE, and regional repositories) may introduce selection bias. The exclusive reliance on English-language publications may have omitted relevant non-English studies from Asian acupuncture research centers, potentially introducing selection bias and limiting the external validity of the findings. Furthermore, the algorithmic dependencies of CiteSpace may have influenced the results. Secondly, the relatively small number of included RCTs reflects the stringent inclusion criteria applied to maintain methodological rigor. This limitation emphasizes the need for additional high-quality RCTs in the field. However, the included studies exhibited notable heterogeneity in quality, driven by variations in design, sample sizes, and measurement standardization, which may affect the integration and comparability of the findings. Lastly, although this research endeavored to forecast future research directions, such predictions were inherently speculative, without establishing definitive causal linkages. Third, our literature search was limited to the Web of Science Core Collection. While this database is widely recognized for bibliometric studies due to its citation indexing and journal coverage, the exclusion of other databases such as PubMed may introduce selection bias. Future updates will incorporate multi-database searches (e.g., PubMed, EMBASE, and regional repositories) to enhance comprehensiveness.

## 6 Conclusion

In summary, this study provided a bibliometric visualization analysis of significant literature in the field of acupuncture therapy for PNSD over the past 20 years, utilizing CiteSpace software. As discussed in previous sections, the findings revealed fundamental insights and identified current hotspots and research frontiers. Although acupuncture therapy appeared to be effective in treating PNSD, there was a notable lack of robust evidence supporting its efficacy. Consequently, further researches, including high-quality RCTs or systematic reviews, should investigate the mechanistic pathways of acupuncture in different PNSD conditions and establish standardized protocols for its application.

## Data Availability

The original contributions presented in the study are included in the article/Supplementary material, further inquiries can be directed to the corresponding authors.

## References

[1] Watson JC Dyck PJB. Peripheral neuropathy: a practical approach to diagnosis and symptom management. Mayo Clin Proc. (2015) 90:940–51. doi: 10.1016/j.mayocp.2015.05.004, PMID: 26141332

[2] Hicks CW Selvin E. Epidemiology of peripheral neuropathy and lower extremity disease in diabetes. Curr Diab Rep. (2019) 19:86. doi: 10.1007/s11892-019-1212-8, PMID: 31456118 PMC6755905

[3] Finnerup NB Kuner R Jensen TS. Neuropathic pain: from mechanisms to treatment. Physiol Rev. (2021) 101:259–301. doi: 10.1152/physrev.00045.2019, PMID: 32584191

[4] Smith TJ Wang EJ Loprinzi CL. Cutaneous Electroanalgesia for relief of chronic and neuropathic pain. N Engl J Med. (2023) 389:158–64. doi: 10.1056/NEJMra2110098, PMID: 37437145

[5] He Y Guo X May BH Zhang AL Liu Y Lu C . Clinical evidence for Association of Acupuncture and Acupressure with Improved Cancer Pain a Systematic Review and Meta-analysis. JAMA Oncol. (2020) 6:271–8. doi: 10.1001/jamaoncol.2019.5233, PMID: 31855257 PMC6990758

[6] Vickers AJ Vertosick EA Lewith G MacPherson H Foster NE Sherman KJ . Acupuncture for chronic pain: update of an individual patient data Meta-analysis. J Pain. (2018) 19:455–74. doi: 10.1016/j.jpain.2017.11.005, PMID: 29198932 PMC5927830

[7] Berman BM Lao LX Langenberg P Lee WL Gilpin AMK Hochberg MC. Effectiveness of acupuncture as adjunctive therapy in osteoarthritis of the knee – a randomized, controlled trial. Ann Intern Med. (2004) 141:901–10. doi: 10.7326/0003-4819-141-12-200412210-00006 15611487

[8] Brinkhaus B Ortiz M Witt CM Roll S Linde K Pfab F . Acupuncture in patients with seasonal allergic rhinitis a randomized trial. Ann Intern Med. (2013) 158:225–34. doi: 10.7326/0003-4819-158-4-201302190-00002, PMID: 23420231

[9] Cheuk DKL Yeung W-F Chung KF Wong V. Acupuncture for insomnia. Cochrane Database Syst Rev. (2012) 2012:CD005472. doi: 10.1002/14651858.CD005472.pub3, PMID: 17636800

[10] Liu Z Yan S Wu J He L Li N Dong G . Acupuncture for chronic severe functional constipation a randomized trial. Ann Intern Med. (2016) 165:761–9. doi: 10.7326/M15-3118, PMID: 27618593

[11] Huang M-C Chang S-C Liao W-L Ke TW Lee AL Wang HM . Acupuncture May help to prevent chemotherapy-induced peripheral neuropathy: a randomized, sham-controlled, single-blind study. Oncologist. (2023) 28:E436–47. doi: 10.1093/oncolo/oyad065, PMID: 36971468 PMC10243779

[12] Yang K Wang Y Li Y-w Chen YG Xing N Lin HB . Progress in the treatment of diabetic peripheral neuropathy. Biomed Pharmacother. (2022) 148:112717. doi: 10.1016/j.biopha.2022.112717, PMID: 35193039

[13] Zhang X-w Hou W-b Pu F-l Wang XF Wang YR Yang M . Acupuncture for cancer-related conditions: an overview of systematic reviews. Phytomedicine. (2022) 106:154430. doi: 10.1016/j.phymed.2022.154430, PMID: 36099656

[14] Coyle ME Liang H Wang K Zhang AL Guo X Lu C . Acupuncture plus moxibustion for herpes zoster: a systematic review and meta-analysis of randomized controlled trials. Dermatol Ther. (2017) 30:e12468. doi: 10.1111/dth.12468, PMID: 28338265

[15] Chen D Zhang G Wang J Chen S Wang J Nie H . Mapping trends in Moyamoya Angiopathy research: a 10-year bibliometric and visualization-based analyses of the web of science Core collection (WoSCC). Front Neurol. (2021) 12:637310. doi: 10.3389/fneur.2021.637310, PMID: 33737903 PMC7960774

[16] Yang W Liu X Zhang X Li C Li Z Li Y . Bibliometric analysis of acupuncture and moxibustion treatment for mild cognitive impairment. Front Neurosci. (2023) 17:1209262. doi: 10.3389/fnins.2023.1209262, PMID: 37397443 PMC10307968

[17] Hu Q Zheng X Li X Liu B Yin C Li Y . Electroacupuncture alleviates mechanical allodynia in a rat model of complex regional pain syndrome type-I via suppressing spinal CXCL12/CXCR4 signaling. J Pain. (2020) 21:1060–74. doi: 10.1016/j.jpain.2020.01.007, PMID: 32006698

[18] Li X Zhi L Han KY Li SQ Ahmad K Seluzicki C . Impact of baseline expectancy on outcome prediction of real and sham acupuncture for persistent chemotherapy-induced peripheral neuropathy pain in solid tumor survivors: a secondary analysis of a randomized clinical trial. Integr Cancer Ther. (2023) 22:15347354221149992. doi: 10.1177/15347354221149992, PMID: 36691937 PMC9893060

[19] Zhang Y Chen R Hu Q Wang J Nie H Yin C . Electroacupuncture ameliorates mechanical allodynia of a rat model of CRPS-I via suppressing NLRP3 Inflammasome activation in spinal cord dorsal horn neurons. Front Cell Neurosci. (2022) 16:826777. doi: 10.3389/fncel.2022.826777, PMID: 35693886 PMC9174662

[20] Sun J Li R Li X Chen L Liang Y Zhang Q . Electroacupuncture therapy for change of pain in classical trigeminal neuralgia study protocol clinical trial (SPIRIT compliant). Medicine. (2020) 99:e19710. doi: 10.1097/MD.0000000000019710, PMID: 32311955 PMC7440061

[21] Mao JJ Ismaila N Bao T Barton D Ben-Arye E Garland EL . Integrative medicine for pain Management in Oncology: Society for Integrative Oncology-ASCO guideline. J Clin Oncol. (2022) 40:3998–4024. doi: 10.1200/JCO.22.01357, PMID: 36122322

[22] Lu W Giobbie-Hurder A Freedman RA Shin IH Lin NU Partridge AH . Acupuncture for chemotherapy-induced peripheral neuropathy in breast Cancer survivors: a randomized controlled pilot trial. Oncologist. (2020) 25:310–8. doi: 10.1634/theoncologist.2019-0489, PMID: 32297442 PMC7160396

[23] Dimitrova A Murchison C Oken B. Acupuncture for the treatment of peripheral neuropathy: a systematic review and Meta-analysis. J Altern Complement Med. (2017) 23:164–79. doi: 10.1089/acm.2016.0155, PMID: 28112552 PMC5359694

[24] Han X Wang L Shi H Zheng G He J Wu W . Acupuncture combined with methylcobalamin for the treatment of chemotherapy-induced peripheral neuropathy in patients with multiple myeloma. BMC Cancer. (2017) 17:17. doi: 10.1186/s12885-016-3037-z, PMID: 28068938 PMC5223334

[25] Molassiotis A Suen LKP Cheng HL Mok TSK Lee SCY Wang CH . A randomized Assessor-blinded wait-list-controlled trial to assess the effectiveness of acupuncture in the management of chemotherapy-induced peripheral neuropathy. Integr Cancer Ther. (2019) 18:1534735419836501. doi: 10.1177/1534735419836501, PMID: 30905173 PMC6434440

[26] Bao T Seidman AD Piulson L Vertosick E Chen X Vickers AJ . A phase IIA trial of acupuncture to reduce chemotherapy-induced peripheral neuropathy severity during neoadjuvant or adjuvant weekly paclitaxel chemotherapy in breast cancer patients. Eur J Cancer. (2018) 101:12–9. doi: 10.1016/j.ejca.2018.06.008, PMID: 30007894 PMC6147260

[27] Kong JT Puetz C Tian L Haynes I Lee E Stafford RS . Effect of Electroacupuncture vs sham treatment on change in pain severity among adults with chronic low Back pain a randomized clinical trial. JAMA Netw Open. (2020) 3:14. doi: 10.1001/jamanetworkopen.2020.22787, PMID: 33107921 PMC7592030

[28] Ursini T Tontodonati M Manzoli L Polilli E Rebuzzi C Congedo G . Acupuncture for the treatment of severe acute pain in herpes zoster: results of a nested, open-label, randomized trial in the VZV pain study. BMC Complement Altern Med. (2011) 11:8. doi: 10.1186/1472-6882-11-46, PMID: 21639941 PMC3125389

[29] Gao XM Wang CY Ni Y Zhang HL. Clinical effect of acupuncture along fascia, meridians, and nerves combined with ultrasound-guided paravertebral nerve block in the treatment of postherpetic neuralgia: a randomized parallel-controlled study. J Tradit Chin Med. (2023) 43:359–64. doi: 10.19852/j.cnki.jtcm.2023.02.007 36994525 PMC10012190

[30] Stringer J Ryder WD Mackereth PA Misra V Wardley AM. A randomised, pragmatic clinical trial of ACUpuncture plus standard care versus standard care alone FOr chemotherapy induced peripheral neuropathy (ACUFOCIN). Eur J Oncol Nurs. (2022) 60:102171. doi: 10.1016/j.ejon.2022.102171, PMID: 35952460 PMC9592667

[31] Chao MT Schillinger D Nguyen U Santana T Liu R Gregorich S . A randomized clinical trial of group acupuncture for painful diabetic neuropathy among diverse safety net patients. Pain Med. (2019) 20:2292–302. doi: 10.1093/pm/pnz117, PMID: 31127837 PMC7963203

[32] Dietzel J Habermann IV Hörder S Hahn K Meyer-Hamme G Ortiz M . Acupuncture in patients with diabetic peripheral neuropathy-related complaints: a randomized controlled clinical trial. J Clin Med. (2023) 12:13. doi: 10.3390/jcm12062103, PMID: 36983105 PMC10055667

[33] Ju ZY Wang K Cui HS Yao Y Liu SM Zhou J . Acupuncture for neuropathic pain in adults. Cochrane Database Syst Rev. (2017) 12:CD012057. doi: 10.1002/14651858.CD012057.pub2 29197180 PMC6486266

[34] Mao JJ Liou KT Baser RE Bao T Panageas KS Romero SAD . Effectiveness of Electroacupuncture or auricular acupuncture vs usual Care for Chronic Musculoskeletal Pain among Cancer Survivors the PEACE randomized clinical trial. JAMA Oncol. (2021) 7:720–7. doi: 10.1001/jamaoncol.2021.0310, PMID: 33734288 PMC7974834

[35] Seo SY Lee K-B Shin J-S Lee J Kim MR Ha IH . Effectiveness of acupuncture and Electroacupuncture for chronic neck pain: a systematic review and Meta-analysis. Am J Chin Med. (2017) 45:1573–95. doi: 10.1142/S0192415X17500859, PMID: 29121797

[36] Ulett GA Han SP Han JS. Electroacupuncture: mechanisms and clinical application. Biol Psychiatry. (1998) 44:129–38. doi: 10.1016/S0006-3223(97)00394-6, PMID: 9646895

[37] Wang Y Li W Peng W Zhou J Liu Z. Acupuncture for postherpetic neuralgia systematic review and meta-analysis. Medicine. (2018) 97:e11986. doi: 10.1097/MD.0000000000011986, PMID: 30142834 PMC6113033

[38] Pei L-X Yi Y Guo J Chen L Zhou JY Wu XL . The effectiveness and safety of acupuncture/electroacupuncture for chemotherapy-induced peripheral neuropathy: a systematic review and meta-analysis. Acupunct Med. (2023) 41:73–85. doi: 10.1177/09645284221076512, PMID: 35695033

[39] Papadopoulou M Stamou M Bakalidou D Moschovos C Zouvelou V Zis P . Non-pharmacological interventions on pain and quality of life in chemotherapy induced polyneuropathy: systematic review and Meta-analysis. In Vivo. (2023) 37:47–56. doi: 10.21873/invivo.13053, PMID: 36593011 PMC9843771

[40] Hu H Chen L Ma R Gao H Fang J. Acupuncture for primary trigeminal neuralgia: a systematic review and PRISMA-compliant meta-analysis. Complement Ther Clin Pract. (2019) 34:254–67. doi: 10.1016/j.ctcp.2018.12.013, PMID: 30712736

[41] Qi T Lan H Zhong C Zhang R Zhang H Zhu F . Systematic review and meta-analysis: the effectiveness and safety of acupuncture in the treatment of herpes zoster. Ann Palliat Med. (2022) 11:756–65. doi: 10.21037/apm-22-109, PMID: 35249352

[42] Ang L Kim H-J Heo J-W Choi TY Lee HW Kim JI . Acupuncture for the treatment of trigeminal neuralgia: a systematic review and meta-analysis. Complement Ther Clin Pract. (2023) 52:101763. doi: 10.1016/j.ctcp.2023.101763, PMID: 37159979

[43] Greenlee H Crew KD Capodice J Awad D Buono D Shi Z . Randomized sham-controlled pilot trial of weekly electro-acupuncture for the prevention of taxane-induced peripheral neuropathy in women with early stage breast cancer. Breast Cancer Res Treat. (2016) 156:453–64. doi: 10.1007/s10549-016-3759-2, PMID: 27013473 PMC4924571

[44] Rostock M Jaroslawski K Guethlin C Ludtke R Schroeder S Bartsch HH. Chemotherapy-induced peripheral neuropathy in Cancer patients: a four-arm randomized trial on the effectiveness of Electroacupuncture. Evid Based Complement Alternat Med. (2013) 2013:1–9. doi: 10.1155/2013/349653, PMID: 24066010 PMC3771477

[45] de Sousa TR Mattos S Marcon G Furtado T Duarte Da Silva M. Acupuncture techniques and acupoints used in individuals under chemotherapy or radiotherapy treatment of cancer: a systematic review. J Clin Nurs. (2023) 32:6917–33. doi: 10.1111/jocn.16812, PMID: 37382085

[46] Zhang T Zhang Q Zhu P Sun W Ding Z Hu L. The efficacy of acupuncture in the treatment of chemotherapy-induced peripheral neuropathy: a network Meta-analysis. Altern Ther Health Med. (2023) 29:898–906. PMID: 37708563

[47] Li K Giustini D Seely D. A systematic review of acupuncture for chemotherapy-induced peripheral neuropathy. Curr Oncol. (2019) 26:E147–54. doi: 10.3747/co.26.4261, PMID: 31043820 PMC6476456

[48] Garcia MK Cohen L Guo Y Zhou Y You B Chiang J . Electroacupuncture for thalidomide/bortezomib-induced peripheral neuropathy in multiple myeloma: a feasibility study. J Hematol Oncol. (2014) 7:7. doi: 10.1186/1756-8722-7-41, PMID: 24886772 PMC4038108

[49] Schroeder S Liepert J Remppis A Greten JH. Acupuncture treatment improves nerve conduction in peripheral neuropathy. Eur J Neurol. (2007) 14:276–81. doi: 10.1111/j.1468-1331.2006.01632.x, PMID: 17355547

[50] Bao T Goloubeva O Pelser C Porter N Primrose J Hester L . A pilot study of acupuncture in treating Bortezomib-induced peripheral neuropathy in patients with multiple myeloma. Integr Cancer Ther. (2014) 13:396–404. doi: 10.1177/1534735414534729, PMID: 24867959 PMC4562796

[51] Li Y Lustberg MB Hu S. Emerging pharmacological and non-pharmacological therapeutics for prevention and treatment of chemotherapy-induced peripheral neuropathy. Cancers. (2021) 13:766. doi: 10.3390/cancers13040766, PMID: 33673136 PMC7918689

[52] Mezzanotte JN Grimm M Shinde NV Nolan T Worthen-Chaudhari L Williams NO . Updates in the treatment of chemotherapy-induced peripheral neuropathy. Curr Treat Options in Oncol. (2022) 23:29–42. doi: 10.1007/s11864-021-00926-0, PMID: 35167004 PMC9642075

[53] Ben-Arye E Hausner D Samuels N Gamus D Lavie O Tadmor T . Impact of acupuncture and integrative therapies on chemotherapy-induced peripheral neuropathy: a multicentered, randomized controlled trial. Cancer. (2022) 128:3641–52. doi: 10.1002/cncr.34422, PMID: 35960141

[54] Hwang M-S Lee H-Y Choi T-Y Lee JH Ko YS Jo DC . A systematic review and meta-analysis of the efficacy of acupuncture and electroacupuncture against chemotherapy-induced peripheral neuropathy. Medicine. (2020) 99:e19837. doi: 10.1097/MD.0000000000019837, PMID: 32332632 PMC7220547

[55] Smith TJ Coyne PJ Parker GL Dodson P Ramakrishnan V. Pilot trial of a patient-specific cutaneous electrostimulation device (MC5-a Calmare®) for chemotherapy-induced peripheral neuropathy. J Pain Symptom Manag. (2010) 40:883–91. doi: 10.1016/j.jpainsymman.2010.03.022, PMID: 20813492 PMC4383258

[56] Pei W Zeng J Lu L Lin G Ruan I. Is acupuncture an effective postherpetic neuralgia treatment? A systematic review and meta-analysis. J Pain Res. (2019) 12:2155–65. doi: 10.2147/JPR.S199950, PMID: 31410050 PMC6643066

[57] Wu C-h Lv Z-t Zhao Y Gao Y Li JQ Gao F . Electroacupuncture improves thermal and mechanical sensitivities in a rat model of postherpetic neuralgia. Mol Pain. (2013) 9:9. doi: 10.1186/1744-8069-9-18, PMID: 23551937 PMC3626545

[58] Ruengwongroj P Muengtaweepongsa S Patumanond J Phinyo P. Effectiveness of press needle treatment and electroacupuncture in patients with postherpetic neuralgia: a matched propensity score analysis. Complement Ther Clin Pract. (2020) 40:101202. doi: 10.1016/j.ctcp.2020.101202, PMID: 32891279

[59] He K Ni F Huang Y Zheng M Yu H Han D . Efficacy and safety of Electroacupuncture for pain control in herpes zoster: a systematic review and Meta-analysis. Evid Based Complement Alternat Med. (2022) 2022:1–11. doi: 10.1155/2022/4478444, PMID: 35832527 PMC9273388

[60] Liu K Zeng J Pei W Chen S Luo Z Lu L . Assessing the reporting quality in randomized controlled trials of acupuncture for postherpetic neuralgia using the CONSORT statement and STRICTA guidelines. J Pain Res. (2019) 12:2359–70. doi: 10.2147/JPR.S210471, PMID: 31534360 PMC6681161

[61] Wang L Qiu L Zheng X Ouyang J Zhang M He L . Effectiveness of electroacupuncture at Jiaji acupoints (EX-B 2), plus moxibustion and intermediate on postherpetic neuralgia: a randomized controlled trial. J Tradit Chin Med. (2020) 40:121–7. PMID: 32227773

[62] Cruccu G Gronseth G Alksne J Argoff C Brainin M Burchiel K . AAN-EFNS guidelines on trigeminal neuralgia management. Eur J Neurol. (2008) 15:1013–28. doi: 10.1111/j.1468-1331.2008.02185.x, PMID: 18721143

[63] Yin Z Wang F Sun M Zhao L Liang F. Acupuncture methods for primary trigeminal neuralgia: a systematic review and network Meta-analysis of randomized controlled trials. Evid Based Complement Alternat Med. (2022) 2022:1–26. doi: 10.1155/2022/3178154, PMID: 35237333 PMC8885188

[64] Ichida MC Zemuner M Hosomi J Pai HJ Teixeira MJ de Siqueira JTT . Acupuncture treatment for idiopathic trigeminal neuralgia: a longitudinal case-control double blinded study. Chin J Integr Med. (2017) 23:829–36. doi: 10.1007/s11655-017-2786-0, PMID: 29080198

[65] Gao J Zhao C Jiang W Zheng B He Y. Effect of acupuncture on cognitive function and quality of life in patients with idiopathic trigeminal neuralgia. J Nerv Ment Dis. (2019) 207:171–4. doi: 10.1097/NMD.0000000000000937, PMID: 30720599

[66] Xu Z Wang X Wu Y Wang C Fang X. The effectiveness and safety of acupuncture for chemotherapy-induced peripheral neuropathy: a systematic review and meta-analysis. Front Neurol. (2022) 13:13. doi: 10.3389/fneur.2022.963358 PMC9574072 36262831

[67] Nicolian S Butel T Gambotti L Durand M Filipovic-Pierucci A Mallet A . Cost-effectiveness of acupuncture versus standard care for pelvic and low back pain in pregnancy: a randomized controlled trial. PLoS One. (2019) 14:e0214195. doi: 10.1371/journal.pone.0214195, PMID: 31009470 PMC6476478

[68] Zhao W Huang H Liu K Wang S Lin S Long W . Acupuncture and Moxibustion for peripheral neuropathic pain: a frequentist network Meta-analysis and cost-effectiveness evaluation. Evid Based Complement Alternat Med. (2022) 2022:1–13. doi: 10.1155/2022/6886465 PMC8942664 35341147

[69] Yeung W-F Chen S-C Cheung DST Wong CKH Chong TC Ho YS . Self-administered acupressure for probable knee osteoarthritis in middle-aged and older adults: a randomized clinical trial. JAMA Netw Open. (2024) 7:e245830. doi: 10.1001/jamanetworkopen.2024.5830, PMID: 38639940 PMC11031685

[70] Goo B Park Y-C Kim E Sung WS Kim EJ Kim JH . Efficacy, safety and cost-effectiveness of thread-embedding acupuncture for adhesive capsulitis (frozen shoulder): a study protocol for a multicenter, randomized, patient-Assessor blinded, controlled trial. J Pain Res. (2023) 16:623–33. doi: 10.2147/JPR.S396264, PMID: 36880027 PMC9984543

[71] Zhang Y-Q Jing X Guyatt G. Improving acupuncture research: progress, guidance, and future directions. BMJ. (2022) 376:o487. doi: 10.1136/bmj.o487, PMID: 35217523

[72] Kutcher AM LeBaron VT. Evaluating acupuncture for the treatment of chemotherapy-induced peripheral neuropathy: an integrative review. West J Nurs Res. (2022) 44:169–79. doi: 10.1177/0193945921992538, PMID: 33559535

[73] Lu Z Moody J Marx BL Hammerstrom T. Treatment of chemotherapy-induced peripheral neuropathy in integrative oncology: a survey of acupuncture and oriental medicine practitioners. J Altern Complement Med. (2017) 23:964–70. doi: 10.1089/acm.2017.0052, PMID: 28661695

[74] Lee J-H Cho TJ Park MG Kim JH Song SK Park SY . Clinical study on concurrent use of electro-acupuncture or Chuna manual therapy with pregabalin for chemotherapy-induced peripheral neuropathy: safety and effectiveness (open-labeled, parallel, randomized controlled trial, assessor-blinded) a study protocol. Medicine. (2020) 99:e18830. doi: 10.1097/MD.0000000000018830 32011497 PMC7220112

[75] Lu C Bao W Deng D Li R Li G Zou S . Efficacy of electroacupuncture with different frequencies in the treatment of chemotherapy-induced peripheral neuropathy: a study protocol for a randomized controlled trial. Front Neurol. (2022) 13:843886. doi: 10.3389/fneur.2022.843886, PMID: 35968286 PMC9366109

[76] Teng C Egger S Blinman PL Vardy JL. Evaluating laser photobiomodulation for chemotherapy-induced peripheral neuropathy: a randomised phase II trial. Support Care Cancer. (2023) 31:11. doi: 10.1007/s00520-022-07463-y, PMID: 36526802 PMC9758032

[77] Schroeder S Meyer-Hamme G Epplée S. Acupuncture for chemotherapy-induced peripheral neuropathy (CIPN): a pilot study using neurography. Acupunct Med. (2012) 30:4–7. 22146780 10.1136/acupmed-2011-010034

