# Supplementary material for: Research trends of acupuncture therapy for painful peripheral nervous system diseases from 2004 to 2023: a bibliometric and meta-analysis
Source: Front Neurol. 2025 Mar 14;16:1510331. doi: 10.3389/fneur.2025.1510331 (PMC11949873; doi:10.3389/fneur.2025.1510331)
Supplement: Supplementary file 1 [file Supplementary_file_1.docx]

Supplementary Material

# Supplementary Figures and Tables

## Supplementary Figures


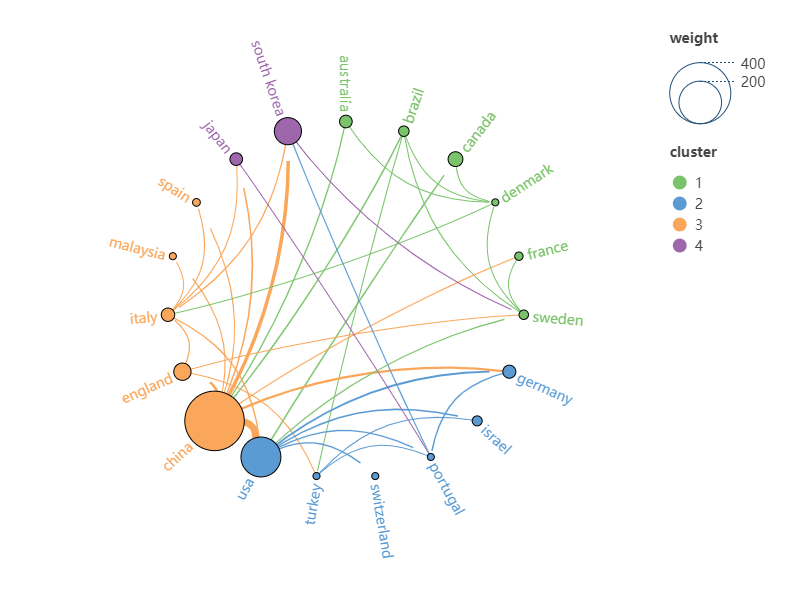


**Supplementary Figure S1.** Cooperation Networks of Countries


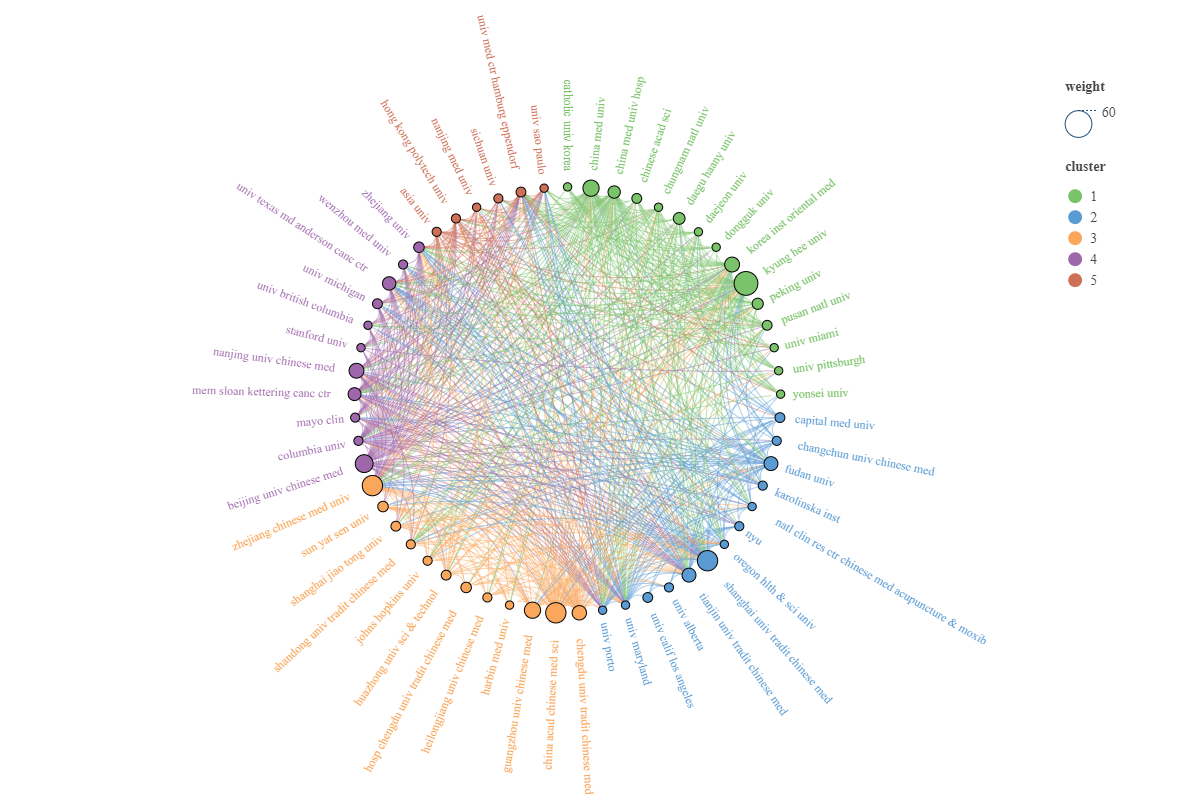


**Supplementary Figure S2.** Cooperation Networks of Institutions


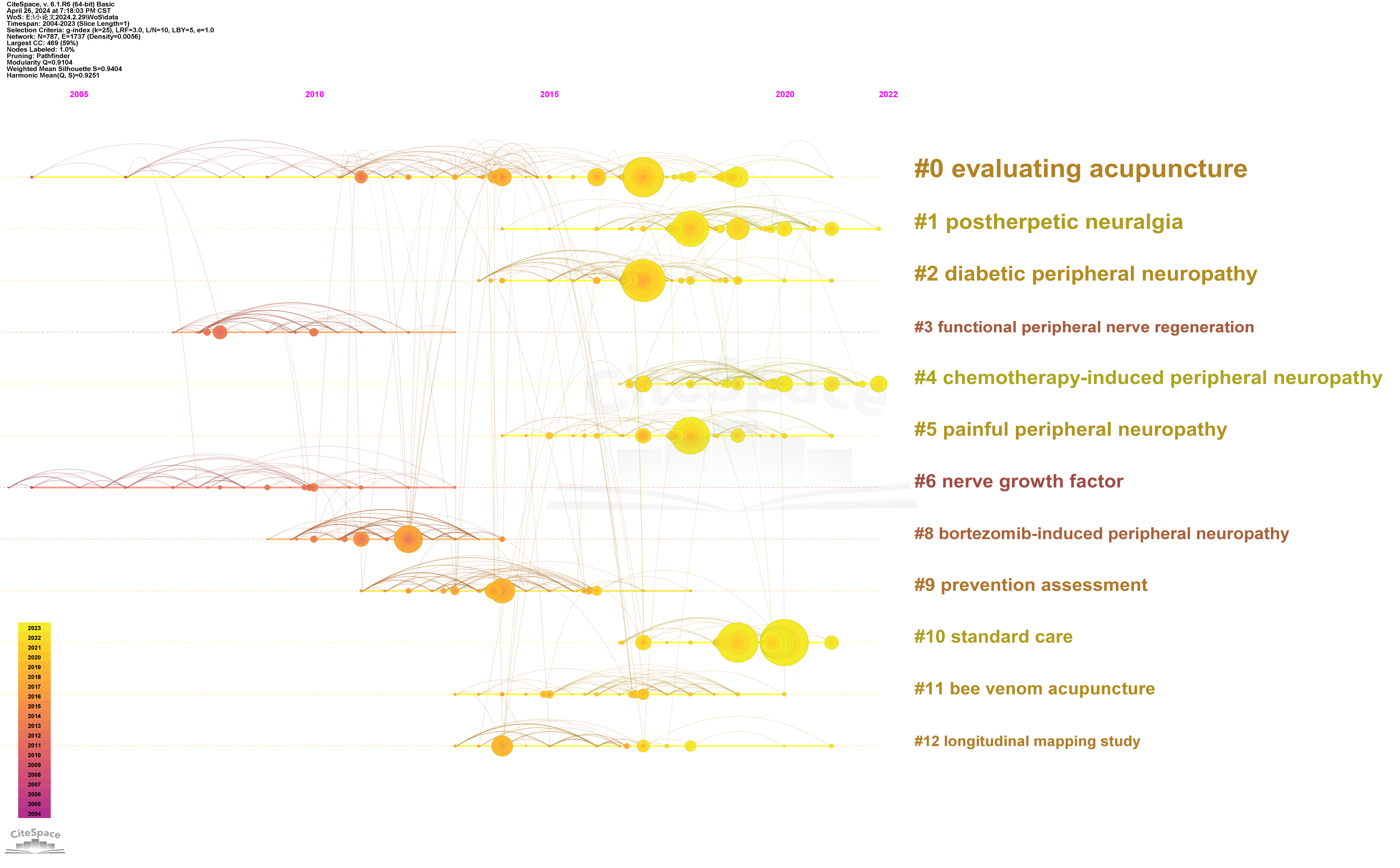


**Supplementary Figure S3.** Timeline view of Co-cited Reference


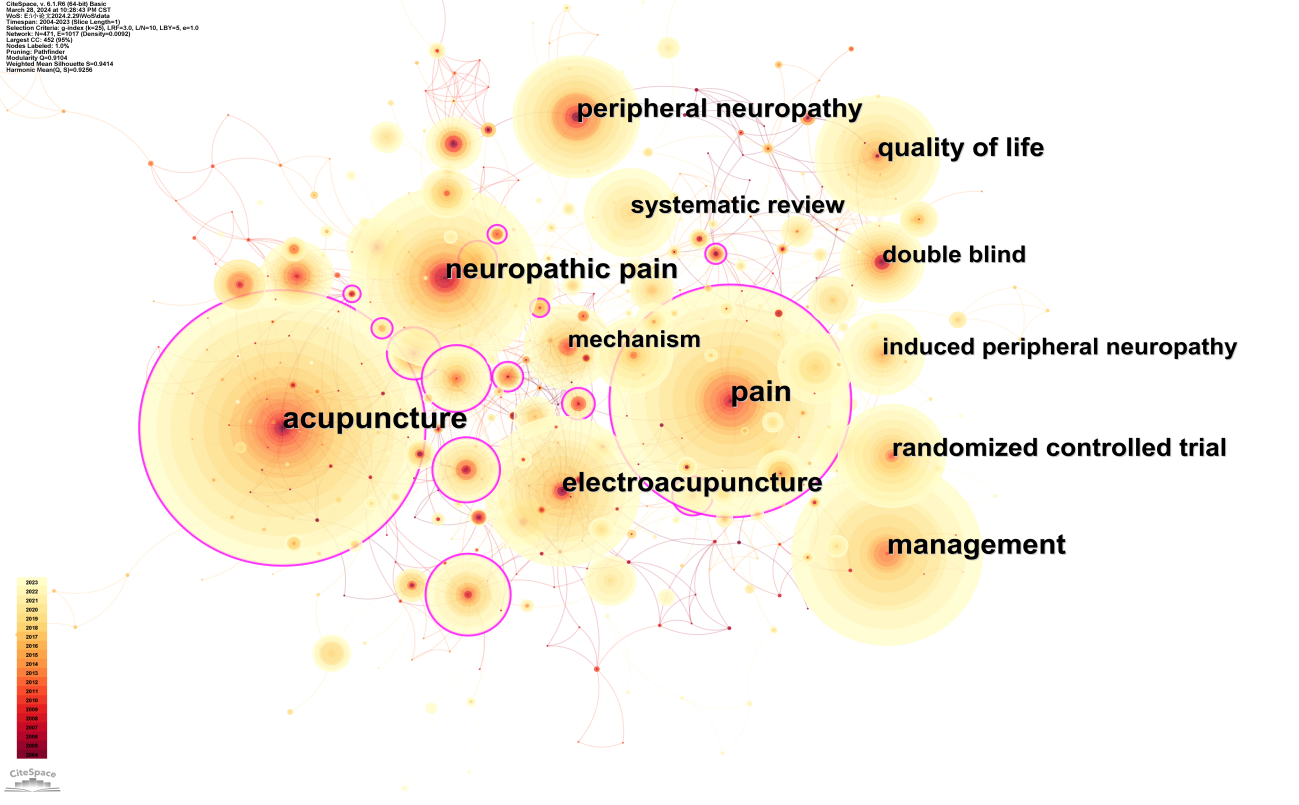


**Supplementary Figure S4.** Collaborative Map of Keywords Co-occurrence

## Supplementary Tables

**Table S1 The Topic Search Query**

| **Set** | **Results** | **Search Query** |
| --- | --- | --- |
| #1 | 22169 | (((((((((TS=(Acupuncture)) OR TS= (Acupuncture Therapy)) OR TS= (Acupuncture Treatment)) OR TS= (Acupotomy)) OR TS= (Acupuncture Analgesia)) OR TS= (Ear Acupuncture)) OR TS= (Electroacupuncture)) OR TS= (Meridians)) OR TS= (Acupuncture Points)) OR TS= (Moxibustion) **Indexes**=SCI-EXPANDED, CCR-EXPANDED, IC **Document Types**=Article or Review Article **Language**=English **Timespan**=2004-2023 |
| #2 | 94505 | ((((((((((((((((((((((((TS=(Peripheral Nervous System Diseases)) OR TS= (PNS Disease)) OR TS= (Peripheral Neuropathy)) OR TS= (Peripheral Nerve Disease)) OR TS= (Peripheral Nervous System Disorders)) OR TS= (Acrodynia)) OR TS= (Amyloid Neuropathies)) OR TS= (Brachial Plexus Neuropathies)) OR TS= (Complex Regional Pain Syndromes)) OR TS= (Diabetic Neuropathies)) OR TS= (Giant Axonal Neuropathy)) OR TS= (Hand-Arm Vibration Syndrome)) OR TS= (Isaacs Syndrome)) OR TS= (Mononeuropathies)) OR TS= (Nerve Compression Syndromes)) OR TS= (Neuralgia)) OR TS= (Neuritis)) OR TS= (Neurofibromatosis 1)) OR TS= (Pain Insensitivity, Congenital)) OR TS= (Peripheral Nerve Injuries)) OR TS= (Peripheral Nervous System Neoplasms)) OR TS= (Polyneuropathies)) OR TS= (Radiculopathy)) OR TS= (Small Fiber Neuropathy)) OR TS= (Tarlov Cysts) **Indexes**=SCI-EXPANDED, CCR-EXPANDED, IC **Document Types**=Article or Review Article **Language**=English **Timespan**=2004-2023 |
| #3 | 678 | #1 AND #2 |

**Table S2 The Top 5 Publications and Centrality of Countries Related to Acupuncture Therapy for PNSD from 2004 to 2023**

| **Rank** | **Publications** | **Country** | **Centrality** | **Country** |
| --- | --- | --- | --- | --- |
| 1 | 355 | China | 0.53 | England |
| 2 | 160 | USA | 0.38 | Italy |
| 3 | 74 | South Korea | 0.37 | Sweden |
| 4 | 28 | England | 0.24 | USA |
| 5 | 22 | Canada | 0.22 | France |

**Table S3 The Top 10 Publications and Centrality of Institutions Related to Acupuncture Therapy for PNSD from 2004 to 2023**

| **Rank** | **Publications** | **Institution** | **Centrality** | **Institution** |
| --- | --- | --- | --- | --- |
| 1 | 40 | Kyung Hee Univ | 0.19 | Univ Texas MD Anderson Canc Ctr |
| 2 | 30 | China Acad Chinese Med Sci | 0.18 | Nanjing Univ Chinese Med |
| 3 | 28 | Zhejiang Chinese Med Univ | 0.18 | Fudan Univ |
| 4 | 28 | Shanghai Univ Tradit Chinese Med | 0.18 | Canadian Coll Naturopath Med |
| 5 | 23 | Beijing Univ Chinese Med | 0.17 | Shanghai Univ Tradit Chinese Med |
| 6 | 19 | Guangzhou Univ Chinese Med | 0.16 | Chang Bing Show Chwan Mem Hosp |
| 7 | 19 | China Med Univ | 0.16 | Changshu 1 Peoples Hosp |
| 8 | 15 | Chengdu Univ Tradit Chinese Med | 0.15 | Asia Univ Hosp |
| 9 | 15 | Korea Inst Oriental Med | 0.12 | McMaster Univ |
| 10 | 14 | Nanjing Univ Chinese Med | 0.1 | Daejeon Univ |

**Table S4 The Top 5 Publications of Authors Related to Acupuncture Therapy for PNSD from 2004 to 2023**

| **Rank** | **Publications** | **Author** |
| --- | --- | --- |
| 1 | 15 | Fang Jianqiao |
| 2 | 10 | Bao Ting |
| 3 | 7 | Kim Sun Kwang |
| 4 | 6 | Ben-arye Eran |
| 5 | 5 | Liang Yi |

**Table S5 The Top 10 Publications of Journals Related to Acupuncture Therapy for PNSD from 2004 to 2023**

| **Rank** | **Publications** | **Journal** | **IF** | **Country** |
| --- | --- | --- | --- | --- |
| 1 | 55 | Medicine | 1.817 | USA |
| 2 | 26 | Evidence-Based Complementary and Alternative Medicine | 2.650 | England |
| 3 | 22 | Acupuncture in Medicine | 1.976 | England |
| 4 | 20 | Journal of Pain Research | 2.832 | England |
| 5 | 14 | Frontiers in Neurology | 4.086 | Switzerland |
| 6 | 13 | Integrative Cancer Therapies | 3.077 | USA |
| 7 | 13 | Trials | 2.728 | England |
| 8 | 12 | Frontiers in Neuroscience | 5.152 | Switzerland |
| 9 | 12 | Journal of Alternative and Complementary Medicine | 2.381 | USA |
| 10 | 10 | Neural Regeneration Research | 6.058 | China |

**Table S6 The Top 5 Frequency and centrality of Cited Journals Related to Acupuncture Therapy for PNSD from 2004 to 2023**

| **Rank** | **Frequency** | **Cited Journal** | **IF** | **Country** | **Centrality** | **Cited Journal** | **IF** | **Country** |
| --- | --- | --- | --- | --- | --- | --- | --- | --- |
| 1 | 386 | Pain | 7.926 | USA | 0.18 | Anesthesia and Analgesia | 6.627 | USA |
| 2 | 290 | Evidence-Based Complementary and Alternative Medicine | 2.650 | England | 0.18 | European Journal of Pharmacology | 5.195 | Netherlands |
| 3 | 260 | Acupuncture in Medicine | 1.976 | England | 0.16 | Nature | 69.504 | England |
| 4 | 200 | Plos One | 3.752 | USA | 0.16 | British Medical Journal | 17.215 | England |
| 5 | 187 | Journal of Pain | 5.383 | USA | 0.15 | Anesthesiology | 9.198 | USA |
